# Sex differences in foraging ecology of a zooplanktivorous little auk *Alle alle* during the pre-laying period: insights from remote sensing and animal-tracking

**DOI:** 10.1186/s12983-024-00534-2

**Published:** 2024-04-17

**Authors:** Dariusz Jakubas, Katarzyna Wojczulanis-Jakubas, Lech Marek Iliszko

**Affiliations:** Department of Vertebrate Ecology and Zoology, Faculty of Biology, University of Gdańsk, Wita Stwosza 59, 80-308 Gdańsk, Poland

**Keywords:** Dive characteristics, Dovekie, Foraging ecology, Pre-incubation period

## Abstract

**Background:**

Energy and time allocation in seabirds differ between consecutive stages of breeding given various requirements of particular phases of the reproductive period. Theses allocations may also be sex-specific considering differential energetic or nutritional requirements of males and females and/or sexual segregation in foraging niches and/or areas. In this study we investigated the foraging ecology of an Arctic, zooplanktivorous seabird, the little auk *Alle alle* during the pre-laying period using remote sensing of the environment and GPS-TDR loggers deployed on birds. We compared foraging trips range and habitats of birds with other stages of the breeding period and between sexes.

**Results:**

We found that little auks during the pre-laying period foraged exclusively in cold sea surface temperature zones (with temperatures < 5 ºC) but in various sea depth zones. They dived to similar depths ranging from -4.0 to -10.9 m, exploring various thermal microhabitats (with mean temperatures values ranging from 2.2 °C in Shelf sea depth zone to 5.9 °C in Deep sea depth zone). The majority of foraging trips and dives characteristics were similar to subsequent phases of breeding. However, home ranges during the pre-laying trips were wider compared to the incubation period. As expected, females exhibited wider foraging niches compared to males (wider range of sea surface temperature and sea depth in foraging locations), which could be explained by sex specific energetic and/or nutritional requirements (females producing an egg). We also delineated local foraging areas important for little auks during their whole breeding season. Protection of these areas is crucial for sustaining the local marine biodiversity.

**Conclusions:**

We found that little auks females during the pre-laying period explored wider foraging niches compared to males. These differences may be attributed to sex-specific nutritional or/and energetical constraints at this stage of breeding. The results of this study also emphasize the importance of shelf Arctic-type water masses as the foraging areas for little auks during successive stages of breeding.

**Supplementary Information:**

The online version contains supplementary material available at 10.1186/s12983-024-00534-2.

## Background

Energy and time allocation in avian parents may greatly differ between consecutive stages of breeding and sexes, due to different parental activities and associated constraints. Parental care, including incubation and chick rearing, is considered as a demanding period for most birds because adults must balance their own energy requirements with those of their offspring [1]. Incubating parents have to transfer warmth to the eggs and guard the nest site which imposes quite a considerable time and energy investment. Then, food provisioning during the chick rearing also requires considerable effort, especially in species foraging on distant and/or unpredictable food resources (e.g. [2, 3]). Sexes may differ in their parental roles, engaging into parental activities with different extent but in the majority of avian species, both parents contribute to parental care [4]. The mating period may also be costly [5, 6] but is rarely considered in the context of parental investment while important to fully understand parental performance [7]. During that period sex differences may be particularly pronounced even in monomorphic and socially monogamous species (i.e. females have to produce eggs, while males guard the nest site and/or fertile mate) [8].

Different demands of various stages of breeding may be expressed in stage-specific foraging tactics and performance (e.g. [9–12]), and that may also be sex-specific. Sex differences in foraging performance, often observed in seabirds species (e.g. [13–16]), have been subjected of a long debate. These differences are sometimes explained in terms of the ‘intersexual competition’ hypothesis that suggests that one sex foraging more efficiently, outcompetes the other [15]. In consequence, sexes utilize different foraging niches or foraging areas [15]. Another explanation of the sex difference in foraging performance is the ‘energetic constraint’ hypothesis that considers sex differences in energetic and/or nutritional requirements at various stages of the breeding cycle [15]. Having different constraints and requirements, males and females target different foraging niches and areas.

Understanding time and energy allocations of seabirds in particular phases of breeding is crucial to understand possible carry-over effects from one phase of breeding to another [17, 18]. In this context, the pre-laying period is particularly important, as it represents initial parental investment; performance at this stage determines the breeding success [15]. For females, effective foraging during this phase is crucial to acquire nutrients for egg production [19, 20]. In some seabird species (procellariforms, alcids), females leave their nesting sites for a period of a few days to several weeks (so called pre-laying exodus), to accumulate resources for egg production [20–23]. For this reason, they may forage at more distant, high quality foraging areas [13, 16, 24–26]. Males, in contrast, often forage closer to the colony as they have to return regularly to the colony to defend the nest site and fertile female (when she comes back from sea) against intruders [23, 27], and in some species also to be ready to take the first long incubation shift [13, 24, 28]. Thus, in many seabirds, trips performed during the pre-laying period are longer in time and distance compared to trips performed in other phases of breeding [16, 25, 26].

Here, we investigated the foraging ecology of a small alcid, the little auk (or dovekie) *Alle alle*. It is a zooplanktivorous seabird breeding colonially in the High Arctic and it is considered the most abundant alcid in the Palearctic [29, 30]. The little auk females lay a single egg annually in a nest usually situated under boulders on a mountain scree. Both partners incubate the egg, then brood (for the few first days) and feed the chick [29]. The sexes are monomorphic in plumage, but males are often bigger than females (though there is a great overlap in measurements between the sexes) [31].

It has been found that little auks foraging is energetically expensive because of the costly types of locomotion both in the air (flapping flight) and in the water (underwater ‘flight’ during diving) [32, 33]. The recent study suggests that the energetic cost of diving could be almost 30% higher than that of flying [34]. To cover their extremely high energetic demand [32, 33] little auks breeding in Svalbard forage almost exclusively on copepods associated with cold Arctic waters (mainly *Calanus glacialis*), which are larger and much richer in energy than their counterparts from warmer Atlantic waters [35–39]. Diet is supplemented by some other zooplanktonic prey [40]. Some of them may be highly energetic (as deep water *Calanus hyperboreus*) or be consumed for nutritional reasons (what maybe especially important for female producing the egg). Foraging ecology of little auks during the chick rearing period is relatively well recognized (e.g. [41–47]). The data from the incubation period are scarce [10] and from the pre-laying period are non-existent.

The cost of egg production in the little auk is assumed to be high, as egg mass constitutes 25–27% of female weight [48] and a re-laid egg (i.e. after a loss) is smaller than the first one (by 2.7%; [49]). However, estimation of energy needed for egg production by female little auk suggests that is not as energetically demanding as it is assumed. It seems that, at least in years when resources are not limited during the egg-laying period, the energetic costs of egg production may be negligible, and then non-resource costs only may affect the female’s body condition [48]. As female little auks probably represent the income breeding strategy (i.e. acquire their energy resources for reproduction concurrently with breeding, without reliance on stores) [50], they are likely to forage extensively before egg laying, to fuel the egg formation. The timing of observed female pre-laying exodus corresponds fairly well with the 5.3 days required for yolk formation [51]. One may expect that special female dietary requirements during egg forming may affect female foraging niches and behaviour.

In this study we investigated foraging ecology of little auks during the pre-laying period. We characterized foraging trips, diving performance and foraging habitats, compared them between the sexes and considered them in respect to other stages of breeding (incubation and chick-rearing).

We formulated the following predictions regarding sex differences in birds foraging performance during the pre-laying period:

1. Assuming special dietary requirements of females producing an egg, and their local acquisition (income breeder strategy), females explore wide areas during the pre-laying period, to collect various prey, rich in unique components, that may be not easily available in closer foraging grounds. Males explore slightly narrower areas, with foraging locations situated closer to the colony as they are constrained by the necessity of nest site defence and fertile female guarding in the colony [27, 51] (and seeking opportunities for extra-pair copulations [27]).
2. Assuming the first expectation to be valid (wider foraging areas of females compared to males and some differences in targeted prey), the sexes differ in foraging trip characteristics, foraging habitat characteristics, and diving profiles.

Additionally, we formulated some predictions regarding differences between the pre-laying period and other phases of breeding.

1. Given little auk food preferences (cold-water zooplankton), they exploit foraging grounds with cold water masses, regardless of breeding stage [10, 41].
2. Considering the necessity of frequent and regular returns to the colony after egg-laying to incubate the egg and feed the chick [52], little auks during the pre-laying period exploit wider home areas performing longer foraging trips compared to subsequent phases of breeding.

## Methods

### Study area

We carried out the study in a large little auks colony on West Spitsbergen, at Ariekammen slopes in Hornsund (77° 00′ N, 15° 33′ E). This area is considered as one of the largest breeding aggregations of this species in Svalbard [53, 54]. The Hornsund area is influenced by both coastal Sørkapp Current, carrying cold, Arctic-type water masses from the northeast Barents Sea and the West Spitsbergen Current (WSC), with warmer Atlantic-type water masses originating from the Norwegian Sea [55, 56]. These two distinct water masses are usually separated by a hydrological front (Arctic or Polar Front) situated on shelf break [57, 58]. The range and distribution of colder and warmer water masses vary considerably among the years [59].

### Fieldwork

To investigate the little auk foraging performance, we used miniature global positioning system (GPS) loggers and GPS-loggers with temperature-depth-records sensor (TDR) produced by ECOTONE (Sopot, Poland). We attached the logger (size 27 × 16 × 12 mm) to the bird’s central back feathers using four transversally applied, 2 mm wide strips of Tesa tape (code 4965, Tesa Tape Inc. Charlotte, NC, USA) at approximately the midpoint of the centre-line of the body. The logger mass of 4.6–4.7 g constituted 2.4–2.8% of body mass of instrumented individuals and was concordant with generally accepted recommendation that the weight of the device should not exceed ca 3% of a bird’s body mass [60]. The loggers used bidirectional radio link with base stations installed in the colony, allowing remote data download, without necessity of bird recapture. Little auks recapture is particularly challenging during the pre-laying period, as birds do not spent much time in the nest, where they could be easily caught. Although we attempt to recapture birds to retrieve the loggers (after charging, they could be re-used), we failed in 75% of cases. Nevertheless, the loggers (attached the way we did) drop off the birds after maximum 4–5 days after the deployment (personal observation), thus birds were burden with the device only for a short period, needed for the study.

During the pre-breeding period we checked daily nests, that were active in previous year/s and other crevices that could be a nest burrow (with pebbles on the ground). In total, we managed to capture eight birds during the pre-laying period (9–12 June 2019, i.e. 9–20 days before the egg laying date in particular nests or median egg laying date for the colony in case of individuals which for we could not find the egg at the spot of the capture). We deployed the loggers on the captured individuals, putting birds back into the nest after no more than 10 min of handling. We instrumented both partners in two nests, and one pair member in the remaining nests.

Of the total of eight loggers (deployed on eight individuals), four were 4 GPS-TDR loggers four 4 GPS-only loggers. Of that, we obtained valuable records (with recorded foraging trips) from six individuals (from four GPS-TDR loggers and two GPS-only loggers). The two other loggers registered only GPS locations from the colony. All individuals were molecularly sexed based on feather samples collected upon capture. In total, we analysed 24 trips of six individuals, in that seven trips of two females and 17 trips of four males (Table 1 and Table S1 in Supplementary Materials) performed during the period from -19 to -5 days before egg laying (Supplementary Materials Fig. S1).

**Table 1:** The numbers and type of deployed loggers and analysed trips of GPS-tracked individuals from the Hornsund colony (SW Spitsbergen) during the pre-laying period in 2019 with their basic statistics. *, **—individuals from the same pair

Bird ID	Logger type	Sex	Maximal distance from colony [km]		Trip Duration [h]		Total Distance Covered [km]		No of Trips
			min	max	min	max	min	max	
SPI01	GPS	F*	3.3	74.6	2.0	84.0	6.8	227.0	4
SPI03	GPS	M*	15.3	112.6	3.4	82.7	30.7	237.7	6
SPI05	GPS-TDR	M	55.7	67.6	12.3	14.0	114.7	152.5	2
SPI06	GPS-TDR	F	57.8	184.1	12.1	50.2	121.6	391.5	3
SPI07	GPS-TDR	M	2.8	103.8	2.8	27.9	5.8	262.8	4
SPI08	GPS-TDR	M**	1.5	38.3	0.6	26.4	3.1	78.2	5
SPI04	GPS	F**	No data collected						
SPI02	GPS	M	No data collected						

To monitor breeding status of logger-equipped individuals, we performed daily controls of the chambers where we captured the birds, starting a day after the logger attachment, and continuing until the end of the egg laying period in the colony. In five of six (83.3%) chambers of instruments birds, we found an incubated egg. The chamber, which we did not find the egg in was a crevice that we suspected to be a nest but that could be also an empty chamber that birds were exploring at the moment of the capture, and then abandoned it. Given that the individual captured there (SPI07) performed trips similar to other individuals (Table 1), we did not exclude it from the analyses.

### Sex identification

We sexed birds in a molecular laboratory at the University of Gdańsk, Poland. We extracted DNA from the basal tip of collected body feathers (as efficient as extraction from avian blood [61]) using a commercial kit, Sherlock AX (A&A Biotechnology, Gdynia, Poland). Of the extracted DNA templates, we amplified introns on the CHD-W and CHD-Z genes located on the sex chromosomes, using the primers F2550 and R2718 in PCR [62] with an annealing temperature of 50 ºC. We visualized the sex differences in the PCR products in UV-light on 1% agarose gel stained in Advanced Midori Green (Nippon Genetics Europe, Düren, Germany). Successful PCR products result in one band (ZZ) for males and two bands for females (ZW), that with 200 bp difference between the length of ZW bands is easily distinguishable. Lengths of both bands were verified in respect to a standard ladder (100–1,000 pb, A&A Biotechnology, Gdynia, Poland).

### GPS and TDR data settings and analyses

We set GPS locations sampling to 15 min. Loggers were activated after the first contact with salt water (i.e. diving) or after 48 h. In GPS-TDR loggers we set measurement interval for pressure and temperature sensor build in housing wall to 2 sec.

We divided raw data from GPS-logger into particular trips using the *track2KBE* package [63] in R software [64]. Based on the GPS locations, we calculated the following metrics for each foraging trip: 1) total distance travelled (km)—the sum of distances between all GPS locations along each individual’s track; 2) maximal range of flight (km) – a straight-line distance from the colony to the most distal point reached on each foraging trip; 3) trip duration (h)—the time interval between colony departure and return for particular individual; 4) stationary locations (hereafter called ‘foraging locations’)—the locations with momentary speed < 15 knots (7.72 m/s; threshold estimated based on momentary speed distribution of all GPS-tracked individuals); such stationary locations suggest foraging behaviour as low transit speed is commonly considered as an indicator of foraging behaviour of marine predators (e.g. [65–67]).

We considered in analyses only trips with > 3 GPS fixes collected and with maximal distance from colony > 2 km. In total, we analysed 24 trips of six GPS-tracked individuals, in that two females and four males (Table 1).

To examine TDR data, we processed raw files in the *diveMov*e package [68] in R software [64]. For dives, we set depth threshold of -0.2 m, i.e. we excluded very shallow dives which were probably not associated with foraging (e.g. bathing, wave induced water splash) from analyses. We used 10 standard metrics generated by the *diveMov*e package and calculated other two: bottom frequency and mean temperature of sensor during the bottom phase of diving. See description of all the variables in Table 2. A dive with high bottom frequency is considered as U-shaped dive while the one with low bottom frequency as V-shaped [46]. V-shaped dives are generally interpreted as ‘searching’ dives (e.g. [69]). If the bird does not encounter a prey, it spends little time on the bottom phase of the dive, resulting in a V-shaped dive profile. Additionally, to compare proportion of V-shaped and U-shaped dives we used a 5 secs threshold of bottom time distinguishing the two types of dives following [14]. We established a 5 secs threshold value based on distribution of bottom time recorded in the studied individuals. See the plot example of a dive with all dive phases and temperature sensor records in Supplementary Materials Fig. S2. To analyze dives in respect to sea depth and sea surface temperature at the foraging grounds, we assign dives to the closest GPS location within a 4 min time window (i.e. 2 min before and after the particular stationary GPS location recorded during foraging trip). Duration of the time window represents quite a conservative approach that maximizes accuracy of the dives locations, while still allowing to get a considerable number of dives (*n* = 51).

**Table 2:** Codes and description of dives variables used in analyses

Short name	Variable	Unit	Description
desctim	descent time	sec	duration of descent phase of dive
botttim	bottom time	sec	duration of bottom phase of dive
asctim	ascent time	sec	duration of ascent phase of dive
divetim	dive time	sec	the whole dive duration
descdist	descent distance	m	the last descent depth reflecting vertical distant of dive
bottdist	bottom distance	m	the sum of absolute depth differences at the bottom phase reflecting the total distance covered at the bottom phase of dive
ascdist	ascent distance	m	the first ascent depth reflecting vertical distant of hauling out
bottdep.median	median of bottom depth	m	median value of depth recorded during bottom phase of dive
maxdep	maximal depth	m	maximum depth recoded during the whole dive
postdive.dur	Post-dive duration	sec	post-dive duration
Temp_sens_mean	Mean sensor temperature	°C	mean temperature of sensor during the bottom phase of diving – variable describing water temperature experienced by little auks during foraging
botfreq	bottom frequency	rate	duration of bottom phase of dive divided by dive duration

### Remote sensing and modelled data

To characterize foraging habitats of little auks we used data on sea surface temperature (SST), chlorophyll a concentration (CHLA), and sea depth. All those features have been recognized as important for the main little auk prey – copepods (e.g. [41, 70–72]). Due to high cloudiness in daily satellite images during the period of GPS-tracking we used 8-days mosaics for period 9–16 June covering majority of the period of GPS-tracking (ranging from 9 to 18 June; Supplementary Materials, Table S1). We extracted SST and CHLA for the foraging locations of little auks from the Moderate-resolution Imaging Spectroradiometer (MODIS) Aqua satellite data. We used the Level 3, 4 km binned data product consisting of an average from all retrieved ‘good’ SST acquired in daytime in channel 11 μm and chlorophyll a data retrieved from reflectance with the use of OC4 algorithm [73]. We extracted sea depth data for the foraging locations from the International Bathymetric Chart of the Arctic Ocean (IBCAO) Version 4.2 with 200 m × 200 m grid cell spacing [74].

To estimate zooplankton biomass on the foraging ground of little auks we used modelled zooplankton biomass in sea water, i.e. mole concentration of zooplankton expressed as carbon in sea water (ZOOC) product from Arctic Ocean Biogeochemistry Analysis and Forecast dataset ARCTIC_ANALYSISFORECAST_BGC_002_004 from Copernicus Marine Service, (10.48670/moi-00003 Accessed on 08 January 2024) for 9 June 2019 (data for that day includes 10-day forecast produced based on the previous day's forecast, thus including the period 9–18 June) with spatial resolution: 6.25 × 6.25 km for -10 m depth layers reflecting mean maximal depth of little auks dives in the studied colony (see details in Results and Discussion).

### Depth, SST and productivity zones

We employed in analyses environmental variables (SST, CHLA, sea depth) as continuous or categorical values (i.e. reclassified into zones with ranges of importance for the most preferred food, cold-water copepods). Zonation allowed us to distinguish microhabitats characterized by various environmental conditions (see details below) and investigate how often they were utilized by little auks. Based on surface sea temperature derived from satellite images we classified foraging environment (given *Calanus glacialis* thermal tolerance) into three thermal zones: 1) Cold water zone – cold water zone with SST up to 5.1 °C; SST < 5.1 °C has been recognized as a range optimal for the Arctic zooplankton community occurring in the Hornsund area [72]; 2) Transitional zone – SST in range from 5.11 °C to 6.0°C; A 6 °C isotherm reflects physiological upper threshold for *Calanus glacialis* functioning in Svalbard [75]; 3) Warm water zone – SST > 6.0 °C representing unfavorable conditions for cold water copepods.

**Fig. 1:**
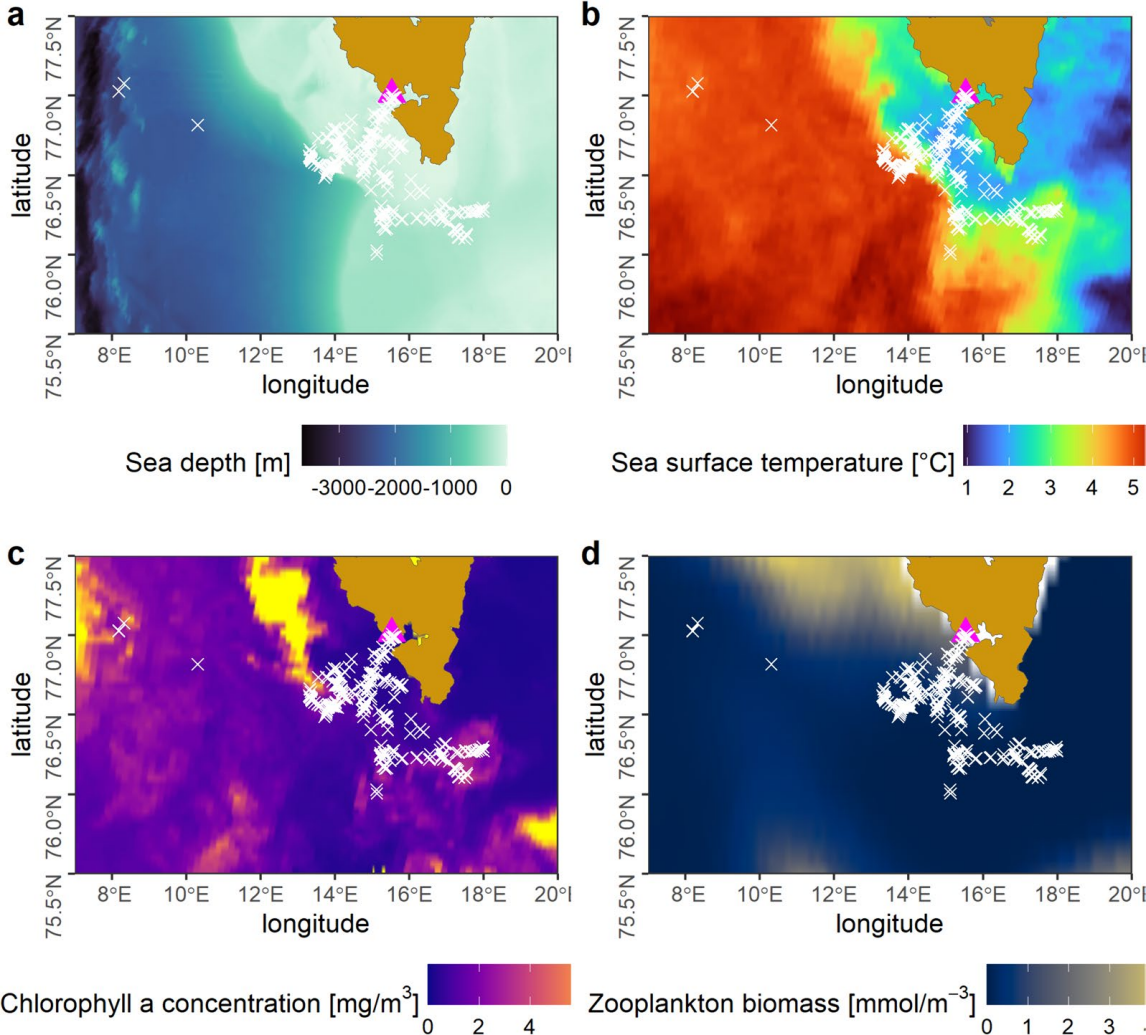
Foraging locations of GPS-tracked little auks during the pre-laying period and environmental conditions in June 2019. **a** Sea depth. **b** Sea surface temperature, **c** Chlorophyll a concentration. **d** Zooplankton biomass. Data sources: MODIS Aqua [73], the International Bathymetric Chart of the Arctic Ocean [74] and Arctic Ocean Biogeochemistry Analysis and Forecast dataset ARCTIC_ANALYSISFORECAST_BGC_002_004 from Copernicus Marine Service, (https://doi.org/10.48670/moi-00003)

Based on data from a bathymetry map we classified foraging environment into three sea depth zones (Fig. 1): 1) Shelf zone with depth from 0 to -242 m; a -242 m isobath has been found to divide Arctic and Atlantic zooplankton communities over shelf and off-shelf zones in the Hornsund area [72]; 2) Off-shelf zone from -243 to -749 m; zone with depth < -242 m has been found to be characteristic for Atlantic zooplankton communities in the Hornsund area [72]; 3) Deep-sea zone with sea depth below -750 m; a -750 m isobath has been used to distinguish deep water zone zooplankton communities in Svalbard by [76];. some prey items preferring this open sea deep water zone in Svalbard (e.g. *Calanus hyperboreus*, *Themisto abyssorum*) were found in the diet of little auk breeding on Spitsbergen [40, 77].

Based on chlorophyll a concentration (CHLA) values we classified four productive regimes following [78, 79]: 1) oligotrophic with low nutrient concentration with CHLA < 0.1 mg/m^3^; 2) mesotrophic with intermediate nutrient concentration with CHLA values 0.1–0.3 mg/m^3^; 3) eutrophic with high nutrient concentration with CHLA values 0.3–1 mg/m^3^; 4) enriched waters with CHLA > 1 mg/m^3^.

In further analyses we also combined sea depth and productive regimes zones finding five Depth-Productivity zones in the studied foraging locations: 1) Deep-Enriched, 2) Off-shelf-Enriched, 3) Off-shelf-Eutrophic, 4) Shelf-Enriched, and 5) Shelf-Eutrophic.

### Statistical analyses

We created maps and visualizations in R software version 4.3.1 [64] or ArcGIS software version 10.3.1 (Redlands, CA, USA: Environmental Systems Research Institute), and performed all statistical analyses in R software version 4.3.1 [64]. For a comparison of GPS tracks of little auks during the pre-laying period investigated in the present study with other phases of the breeding season, we utilized adequate data from other years from the same colony, already published in: [10] for the incubation period (year 2012), and [41] for the chick-rearing period (years 2016 and 2018). We treated chick rearing years independently in the analyses due to differences in environmental conditions between the years when data were collected [43]. In the case of TDR data, we compared them with adequate data collected in the same colony in 2018 during the chick rearing period (unpublished material). Due to assumed sex differences in energy- and time-budget during the pre-laying period, we considered sex in the detailed analysis of the birds foraging performance in that period.

We calculated core and home ranges using autocorrelated kernel densities (aKDE) using the *ctmm* package [80] based on stationary, i.e. foraging locations. The aKDE family of estimators was designed to deal with the complexities of modern movement data: autocorrelation, small sample sizes and missing or irregularly sampled data [81]. As the *ctmm* package calculated autocorrelated kernel densities for particular individuals, we used the function *mean* to calculate mean ranges for sexes or/and periods (for all individuals of both sexes combined). We calculated overlap of aKDEs using Bhattacharya’s affinity (BA) algorithm, a statistical measure of affinity between two populations. This measure ranges from zero (no overlap) to 1 (identical utilization density) [82]. We compared mean core and home ranges areas, maximal ranges of foraging trips, and maximal distance covered during the foraging trip between the pre-laying period and subsequent phases of breeding using permutational analysis of variance (PANOVA; a non-parametric statistical permutation test used to compare groups of objects and test the null hypothesis that the centroids and dispersion of the groups are equivalent for all groups; the significance is computed by permutation of group membership [83]) using the *permuco* package [84].

We also delineated area utilized by little auks from the studied colony in Hornsund during the whole breeding season. We did it by intersecting home ranges for pre-laying, incubation and chick-rearing periods. That resulted in a polygon overlapping home ranges, common for all phases of breeding, and parts of ranges unique for the particular phases.

We compared diving characteristics between periods, sexes and depth zones using mixed permutational analysis of variance (MPANOVA) using the *permuco* package [84]. We used this analysis instead of linear mixed model because of non-normal and multimodal distribution of many variables (even after transformations). We performed separate MPANOVAs for the 12 dive variables (see details in Table 2), with a dive variable as a response variable, sex or period (pre-laying vs. chick-rearing) as an explanatory variable, and foraging trip identity as a random effect (because during one trip birds performed multiple dives). We were not able to use the second random effect (bird identity) because MPANOVA is able to handle only one random effect. To calculate p value we used *Rd_kheradPajouh_renaud* method and 5,000 permutations [84].

We compared the maximal range of foraging trips, total distance covered and total duration of foraging trips between the sexes using MPANOVA with bird identity as a random factor. In case of inter-sex and inter-phase comparisons of distance from the colony to foraging locations, we used MPANOVA with foraging trip identity as a random effect as during one trip birds reached many stationary/foraging locations.

We compared foraging habitat niches of little auks during the pre-laying period (described by SST, CHLA and sea depth in foraging locations of GPS-tracked individuals) between sexes using the *nicheROVER* package [85]. We generated foraging habitat niches using Bayesian analysis of SST, CHLA, and sea depth values at 1,000 runs with a probability level of alpha 0.95. We plotted a random 10 niche regions to create 2-dimensional niche projections. We calculated the size of the foraging habitat niche based on the parameters μ and Σ in a Bayesian context. This allowed to calculate the probability of individual from one sex falling within the niche of another sex [85]. We also compared environmental conditions in foraging locations between males and females using MPANOVA with trip identity as a random factor.

We compared proportions of foraging locations in various Depth and Depth-productivity zones, and various types of dives using chi-squared tests or Fisher’s exact test (in the case of small sample size).

## Results

### Foraging ecology during the pre-laying period

We found that the little auks during the pre-laying period performed foraging trips with maximal ranges from 1.5 to 184.1 km (median 54.5 km, 25–75% percentiles:15.2–78.9 km), with the total distance covered by birds ranging from 3.1 to 391.5 km (median 113.1 km, 25–75% percentiles: 30.5–206.6 km), and the total trip duration ranging from 0.6 to 84.0 h (median 15.1 h, 25–75% percentiles: 6.5–26.3 h) (*N* = 24 trips of six individuals; Table 1).

Foraging locations of GPS-tracked little auks (*N* = 303) were located from 2.6 to 133.7 km from the colony (median 57.3 km, IQR = 32.4, mean ± SD: 59.3 ± 27.58 km). All foraging locations were recorded in Cold SST zone with SST ranging from 1.8 to 5.1 °C (median 2.8 °C, IQR = 1.39, mean ± SD: 3.1 ± 1.01 °C) with prevalence of areas with SST in range between 2 °C and 3.5 °C but with some locations in zone with temperature ~ 5 °C (Fig. 1 and Fig. S3B in Supplementary Materials). Sea depth values in foraging locations ranged from -17 to -2,121 m (median -207 m, IQR = 163, mean ± SD: -288.0 ± 278.18 m). Little auks generally foraged in sea depth from 0 to -400 m with some local peaks in deeper waters (~ 600 m and ~ 1,000 m) (Fig. 1 and Fig. S3A in Supplementary Materials) with 61.4% of locations situated in Shelf zone, 28.8% in Off-shelf zone and only 9.8% in Deep-sea zone (Fig. 1 and S3 in Supplementary Materials). Little auks foraged in locations with chlorophyll a concentration ranging from 0.31 to 4.10 mg · m^−3^ (median 0.91 mg · m^−3^, IQR = 162.0, mean ± SD: 1.18 ± 0.92 mg · m^−3^). Regarding productivity regimes 56.8% locations were located in eutrophic waters and 43.2% in enriched waters (Fig. 1 and S3 in Supplementary Materials). Considering Depth-Productivity zones little auks foraged mainly in Shelf-Eutrophic waters (51.5% records, *N* = 303) and Off-shelf-Enriched waters (23.8). Deep-Enriched, Shelf-Enriched, and Off-shelf-Eutrophic waters comprised 9.9%, 9.6%, and 5.3%, respectively (Supplementary Materials Fig. S4). Zooplankton biomass ranged from 0.0002 to 2.0 mmol · m^−3^ (median 0.14 mmol · m^−3^, IQR 0.44, mean ± SD: 0.43 ± 0.58 mmol · m^−3^). Comparison of zooplankton biomass among Depth-Productivity zones revealed the highest values for Shelf-Eutrophic zone, the most frequently explored by the studied birds. Biomass at this zone was significantly higher than in other zones (Wilcoxon test, *p* < 0.01) (Supplementary Materials Fig. S4).

**Fig. 2:**
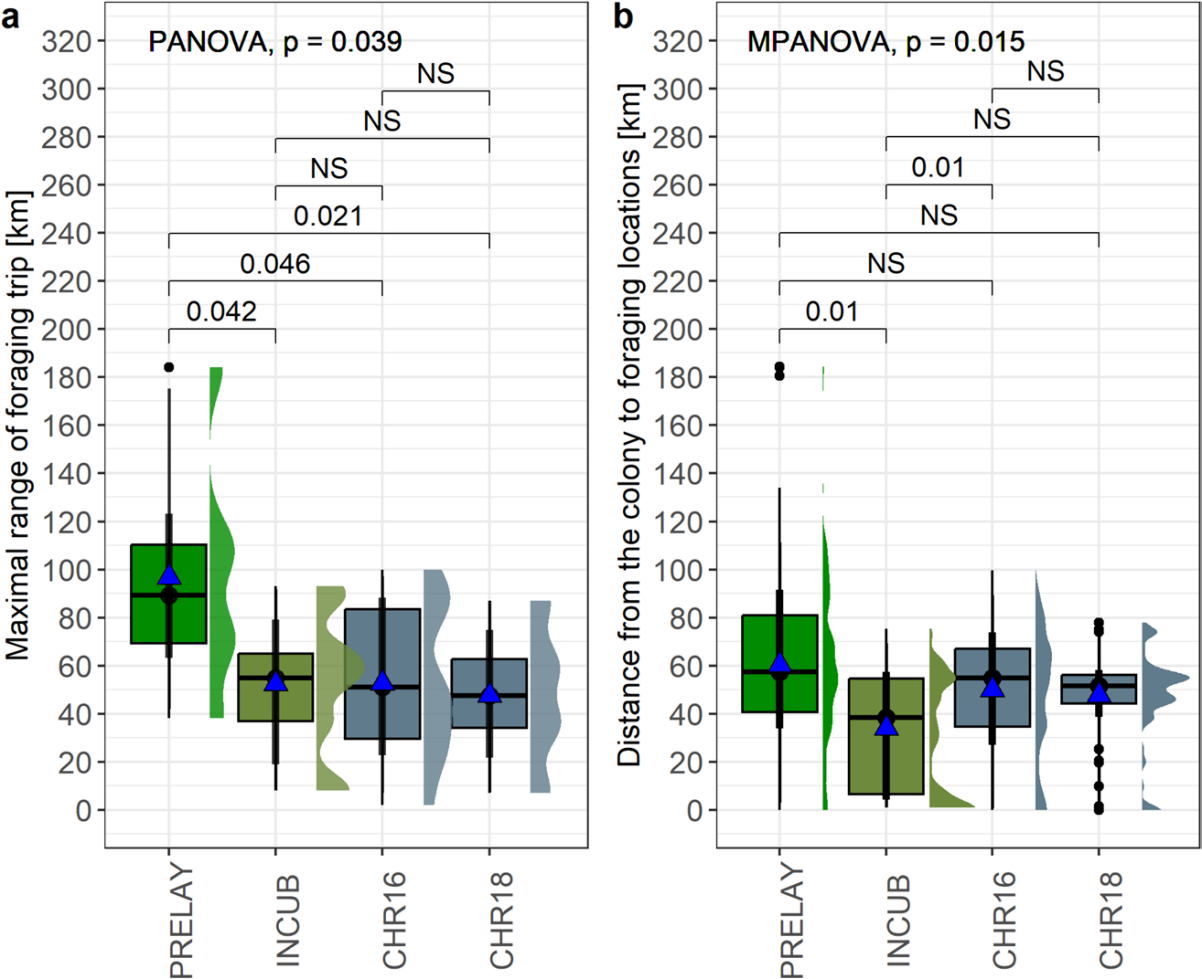
Comparison of foraging trip variables of GPS-tracked little auks between pre-laying (PRELAY), incubation (INCUB) and chick rearing periods in 2016 and 2018 (CHR16 and CHR 18). **a** Maximal range of foraging trips. **b** Distance from the colony to the foraging locations. Boxplots show the median (band inside the box), the first (25%) and third (75%) quartile (box), the lowest and the highest values within 1.5 interquartile range (whiskers), and outliers (dots). Blue triangles indicate mean values. Horizontal lines above boxplots show resampled *p* values from PANOVA or MPANOVA. Right side density plots show distribution of data. NS – not significant differences (*p* > 0.05)

Maximal range of foraging trips (per individual) differed significantly between the phases of breeding season (PANOVA, F = 3.04, resampled *p* = 0.039) with values recorded during the pre-laying period (mean ± SD: 96.8 ± 50.4 km, *N* = 6 individuals) being significantly higher compared to incubation (52.4 ± 30.1 km, *N* = 9 individuals), chick-rearing period in 2016 (52.8 ± 32.2 km, *N* = 11 individuals) and 2018 (47.6 ± 28.4 km, *N* = 10 individuals) (Fig. 2A). However, maximal total distance covered during foraging trip (per individual) did not differ between the studied phases of breeding period (PANOVA, F = 2.388, resampled *p* = 0.091). Distance from the colony to foraging locations differed between particular periods of the breeding season (MPANOVA, F = 3.908, resampled *p* = 0.015). It was higher during the pre-laying period (mean ± SD: 60.3 ± 31.2 km, *N* = 310 locations) but only compared to the incubation period (34.0 ± 23.7 km, *N* = 172 locations; MPANOVA, F = 7.325, resampled *p* = 0.011) (Fig. 2B).

**Fig. 3:**
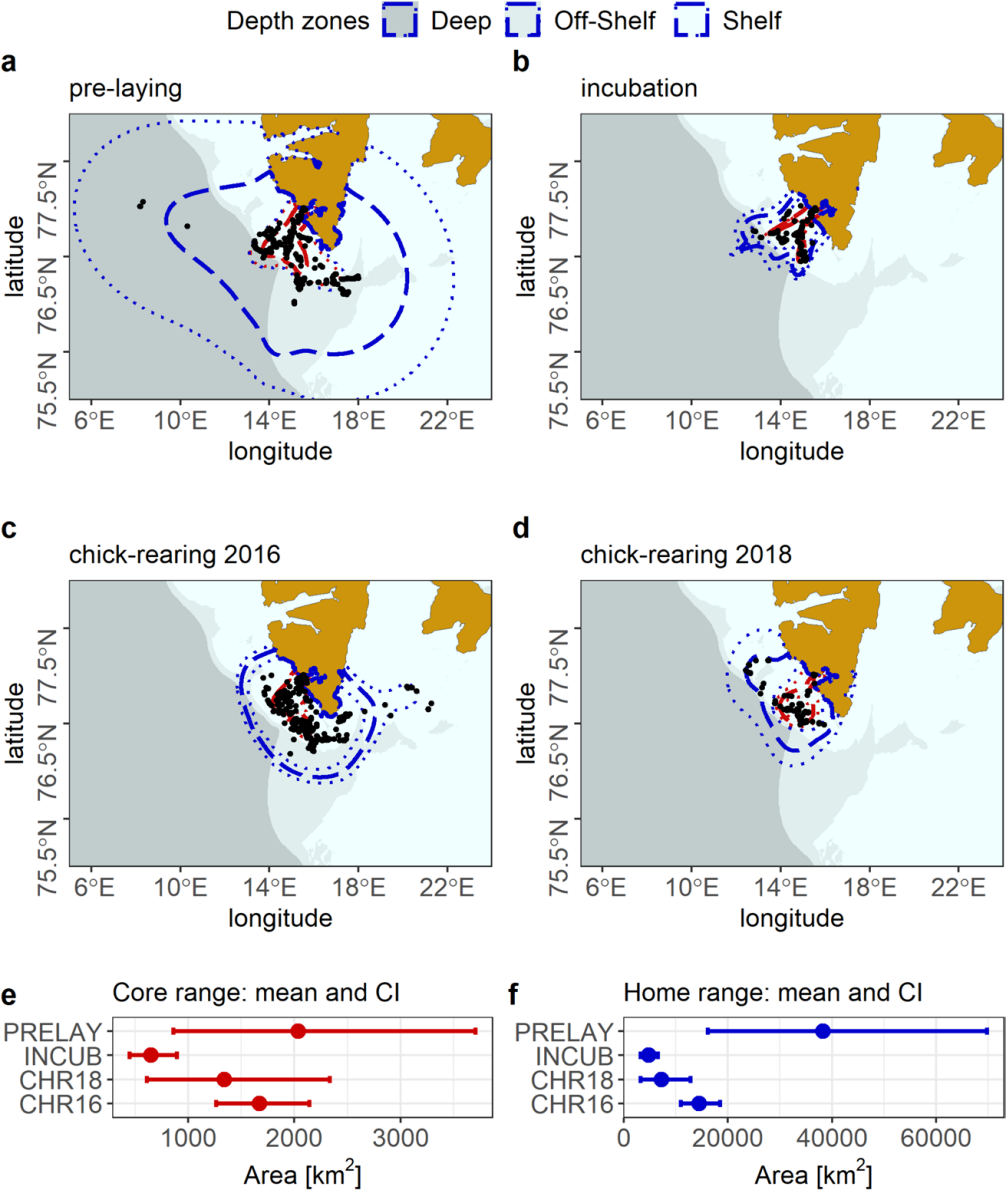
Home and core ranges [autocorrelated 95% and 50% kernel densities (aKDE), respectively] determined based on foraging locations of GPS-tracked little auks in consecutive stages of breeding. **a** Pre-laying 2019 **b** Incubation 2012 **c** Chick-rearing 2016 **d** Chick-rearing period 2018 **e** Mean values and confidence intervals (CI) for home range area **f** Mean values and confidence intervals (CI) for core range area. Blue lines – home range (95% aKDE), red lines—core range (50% aKDE), dashed lines—mean estimate; dotted lines – low and high estimates; black points – foraging locations of little auks. Background: sea depth zones based on isobaths from the International Bathymetric Chart of the Arctic Ocean [74]

Areas of mean home and core ranges of all individuals varied among subsequent phases of breeding (Fig. 3). However, areas of core ranges (50% aKDE) were similar in all studied periods (PANOVA for all individuals combined, F = 1.822, resampled *p* = 0.178). Only areas of home ranges (95% aKDE) differed between periods (PANOVA, F = 3.262, resampled *p* = 0.021) with ranges during the pre-laying (mean ± SD: 38,273 ± 44,409 km^2^) being significantly greater compared to incubation (4,806 ± 1,969 km^2^; PANOVA, F = 4.66, resampled *p* = 0.010), and tending to be greater than during the chick rearing period in 2016 (7,309 ± 2,986 km^2^; PANOVA, F = 2.633, resampled *p* = 0.070), but not significantly different from the chick rearing period in 2018 (14,487 ± 4,938 km^2^; PANOVA, F = 1.862, resampled *p* = 0.200).

Dives of little auks during the pre-laying period (*N* = 4 individuals) were characterized by mean ± SD maximal depth 9.4 ± 7.12, total duration 37.7 ± 19.91 s with bottom phase duration 6.5 ± 6.13 s and bottom phase sensor temperature 3.6 ± 1.60 °C (Table 3). Comparison of various dive variables between pre-laying (*N* = 4,226 dives of 4 individuals) and chick rearing (*N* = 4,357 dives of 7 individuals) periods revealed significant differences (MPANOVA, resampled *p* < 0.02) only for mean sensor temperature during the bottom phase, and bottom frequency with lower mean values recorded during the pre-laying period. Other variables were similar in both phases of breeding (MPANOVA, resampled *p* > 0.12) (Table 3).

**Table 3:** Comparison of diving characteristics of TDR logger equipped little auks between the pre-laying PRLAY and chick-rearing CHR periods. Difference – results of mixed permutational analysis of variance comparing values for PRLAY and CHR. Diving variables description – see Table 1. Significant differences (MPANOVA, resampled *p* < 0.05) are bolded

	No of dives		Mean		Standard deviation		Difference	
Variable	PRLAY	CHR	PRLAY	CHR	PRLAY	CHR	F	p
desctim	4,226	4,357	13.8	18.9	8.87	54.29	0.76	0.440
botttim	3,991	4,165	6.5	16.0	6.13	41.69	2.75	0.127
asctim	4,226	4,357	17.8	22.6	10.65	34.10	1.27	0.297
divetim	4,226	4,357	37.7	56.8	19.91	109.07	1.94	0.207
descdist	4,226	4,357	9.2	8.6	7.07	6.59	0.55	0.457
bottdist	3,991	4,165	1.2	2.6	1.66	2.42	2.75	0.140
ascdist	4,226	4,357	9.0	8.3	6.98	6.63	0.89	0.361
bottdep.median	3,991	4,165	9.3	8.4	6.90	6.54	1.31	0.265
maxdep	4,226	4,357	9.4	9.0	7.12	6.63	0.36	0.548
postdive.dur	4,226	4,357	131.6	81.3	687.53	485.65	2.24	0.169
temp_sens_mean	4,226	4,357	3.6	4.3	1.60	0.94	6.52	**0.015**
botfreq	3,991	4,165	0.2	0.3	0.15	0.19	4.52	**0.0002**

Analyses of pre-laying dives with assigned coordinates (*N* = 51 dives of six individuals) revealed that all were located only in Cold SST zone, with surface water temperature below 5.1 °C (Supplementary Materials Fig. S5A) but in various sea depth zones (Supplementary Materials Fig. S5B). All considered diving variables (maximal depth, dive time, ascent time, descent time, bottom time, bottom distance, bottom frequency, post-dive duration) were similar in all sea depth zones (MPANOVA, resampled *p* > 0.19) except for the mean sensor temperature during the bottom phase of diving (MPANOVA, F = 33.68, resampled *p* = 0.0002) (Supplementary Materials Fig. S5D). Mean sensor temperature at the bottom phase of dive in Shelf zone (mean ± SD: 2.2 ± 0.67 °C, *N* = 33 dives) was significantly lower than in Off-shelf zone (3.9 ± 1.24 °C, *N* = 8 dives) and Deep zone (5.9 ± 0.14 °C, *N* = 10 dives). Sensor temperature in Off-Shelf and Deep sea depth zones did not differ significantly (Supplementary Materials Fig. S5B). Dives of little auks in different sea depth zones were characterized by similar median depths at the bottom phase of diving (means from -4.0 to -10.9 m) (Supplementary Materials Fig. S5D). Also the proportion of V-shaped and U-shaped dives did not differ significantly between the sea depth zones (Fisher’s exact test with simulated p-value based on 2,000 replicates, *p* = 0.599).

### Sex differences

Foraging trips of females (mean ± SD: 70.5 ± 63.2 km, *N* = 7 trips of two individuals) and males (47.7 ± 36.2 km, *N* = 17 trips of four individuals) during the pre-laying period did not differ significantly (MPANOVA) in maximal distance from the colony (F = 0.603, resampled *p* = 0.485). Total distance covered by females (174 ± 142 km) and males (106 ± 86 km) did not differ significantly (F = 1.312, resampled *p* = 0.311). Total trip duration in females (28.5 ± 29.6 h) was similar to males (19.1 ± 19.7 h; F = 1.524, resampled *p* = 0.282). Also the distance from the colony to foraging locations was similar in females (mean ± SD: 62.9 ± 32.5 km, *N* = 157) and males (58.0 ± 29.8 km, *N* = 153) (MPNANOVA, F = 0.119, resampled *p* = 0.729).

**Fig. 4:**
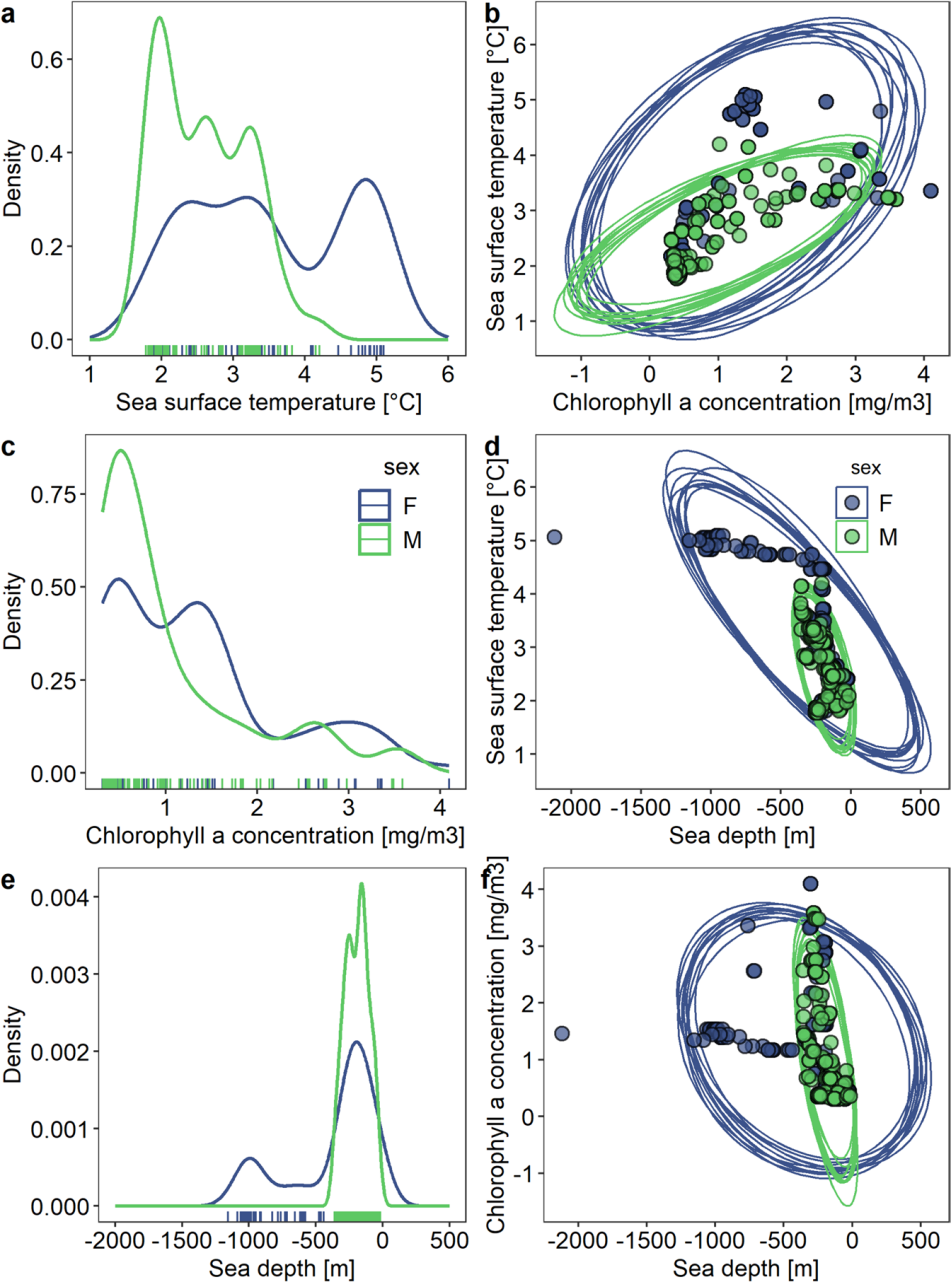
Foraging habitat niches (expressed by sea surface temperature, chlorophyll a concentration, and sea depth in foraging locations of GPS-tracked individuals) of females (blue, *n* = 2) and males (green, *n* = 4) of little auks during the pre-laying period. **a**,** c**,** d** Distribution of particular habitat variables are shown in one-dimensional density plots. **b**, **d**, **f** Two-dimensional scatterplots with ellipses representing ten random projections of the foraging niches in two-dimensional perspectives of two variables

Foraging niches described by SST, CHLA and sea depth differed between sexes (all individuals combined; Fig. 4) and particular individuals (Supplementary Materials, Table S2 and Fig. S6). Females had niches of bigger size (17,398.47 ± SE 2,196.54) than males (1,719.43 ± SE 215.27). Thus, probability of female to be found in male niche (alpha = 95%) was low (24.8%), while the chance for male to fall within female’s niche was very high (98.1%). The largest sex differences in niche size were observed for SST and sea depth (Fig. 4). Indeed, SST values in foraging locations of females (mean ± SD: 3.5 ± 1.10 °C) were significantly higher compared to males (2.6 ± 0.62 °C; MPANOVA, F = 9.689, resampled *p* = 0.006). Females visited during the foraging trips areas with significantly higher sea depth (-381 ± 360 m) compared to males (-194 ± 86.6 m; MPANOVA, F = 4.927, resampled *p* = 0.046). The proportion of foraging locations in particular sea depth zones differed significantly between sexes (χ^2^ test for independence, χ^2^_2_ = 33.085, *p* < 0.001). Only females foraged in Deep sea depth zone and locations from this zone accounted for 19.7% of all recorded female locations. In turn, the proportion of foraging locations of males in Off-shelf and Shelf sea depth zones (i.e. 32.5% and 67.5%, respectively) were higher compared to females (25.7% and 54.6%, respectively) (post-hoc χ^2^ tests, all *p* < 0.001). Females also foraged in locations characterized by higher CHLA values (1.3 ± 0.94 mg/m^3^) compared to males (1.0 ± 0.87 mg/m^3^), however, these differences were not significant (MPANOVA, F = 0.7587, resampled *p* = 0.389). The proportion of foraging locations in particular productivity regimes differed significantly between sexes (χ^2^ test for independence with Yates' continuity correction, χ^2^_2_ = 13.826, *p* = 0.0002). Females foraged more frequently in enriched waters (54.6%) compared to males (45.4%). Males foraged more frequently in eutrophic waters (68.2%) than females (45.4%). Also the proportion of foraging locations in particular Depth-Productivity zones differed significantly between the sexes (χ^2^ test with simulated p-value based on 2,000 replicates, χ^2^ = 40.802, *p* = 0.0005). Both sexes foraged mainly in Shelf-Eutrophic waters [43% of location for females (*n* = 152) and 60% for males (*N* = 151)] and Off-shelf-Enriched waters (24% of location for both sexes). Only females foraged in Deep-Enriched waters (20% female locations), thus, proportion of both sexes differed significantly in all other water types (post-hoc χ^2^ test, all p adjusted for multiple comparisons < 0.0008). Proportion of both sexes also differed significantly between Off-shelf-Eutrophic (with 2% females locations and 9% of males locations) and Shelf-Enriched (with 11% females locations and 8% of males locations) waters (post-hoc χ^2^ test, p adjusted for multiple comparisons = 0.047).

Zooplankton biomass in foraging position visited by females (0.40 ± 0.56 mmol · m^−3^) and males (0.45 ± 0.60 mmol · m^−3^) were similar (MPANOVA, F = 0.049, resampled *p* = 0.083).

**Fig. 5:**
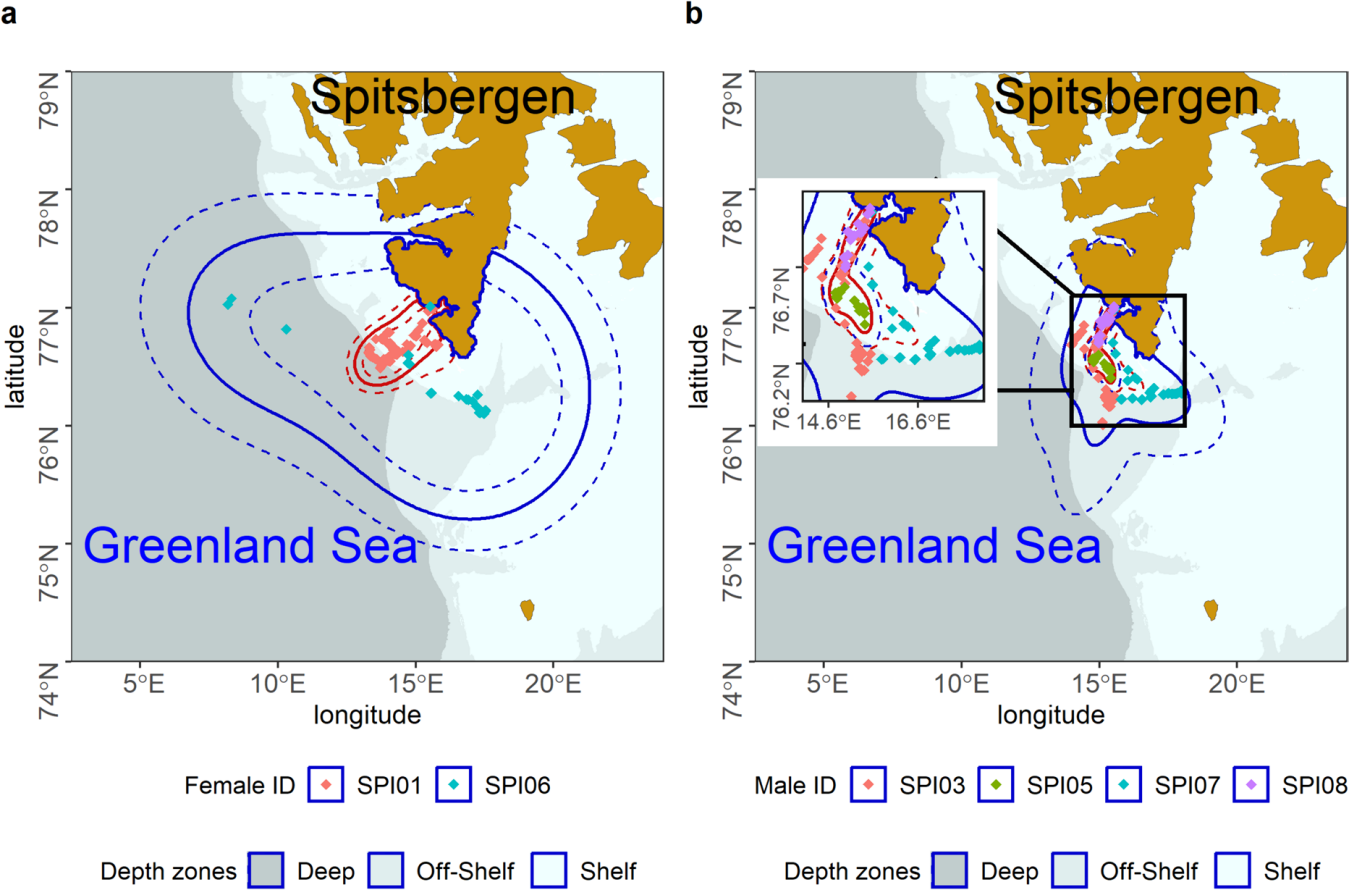
Home and core ranges [autocorrelated 95% and 50% kernel densities (aKDE), respectively] determined based on foraging locations of GPS-tracked little auks during the pre-laying period in 2019. **a** Females (*n* = 2). **b** Males (*N* = 4). Solid lines—mean estimate Dashed lines – low and high estimates; blue lines – home range (95% aKDE), red lines—core range (50% aKDE); points – foraging locations. Inlet – zoom of core range for males. Background: sea depth zones: Deep, Off-shelf and Shelf

Home ranges of females (mean 68,274 km^2^; CI: 39,815 – 104,281 km^2^, *N* = 2 individuals) were larger compared to males (mean: 9,945 km^2^; CI: 1,574 – 25,875 km^2^, *N* = 4 individuals), however, not significantly (PANOVA, F = 2.593, resampled *p* = 0.20). Also core ranges in females (3,893 km^2^; CI: 2,288 – 5,921) were larger than in males (862 km^2^; CI: 508 – 1,308 km^2^) but also not significantly (PANOVA, F = 2.607, resampled *p* = 0.20). Mean inter-individual overlap in home ranges, expressed as the 95% Bhattacharyya’s affinity (BA), was 0.54 for females-males comparison and was higher than for females-females and males-males comparison (BA = 0.48 for both). Males ranges were restricted mainly to shallow Shelf depth sea zone; females ranges included all sea depth zones (Fig. 5).

We found significant inter-sex differences (MPANOVA, *p* ≤ 0.002) in majority of dives variables except for post-dive duration and bottom frequency (*p* > 0.12) (Table 4). In variables differing significantly between sexes mean values for males were higher than in female, except for the mean sensor temperature with the opposite pattern (Table 4). The proportion of V- and U-shaped dives distinguished based on bottom duration differed significantly between sexes (χ^2^ test with Yates' continuity correction, χ^2^_1_ = 254.04, *p* < 0.0001) with female performing more V-shaped dives (71.6% of 1,742 dives) compared to males (46.4% of 2,249 dives).

**Table 4:** Inter-sex comparison of dives characteristics of TDR logger equipped little auks (one female and three males) during the pre-laying period. *P* value – results of mixed permutational analysis of variance. Diving variables description – see Table 1. Significant differences (MPANOVA, resampled *p* < 0.05) are bolded

	No of dives		Mean		Standard deviation		Difference	
Variable	Females	Males	Females	Males	Females	Males	F	p
desctim	1,853	2,373	10.6	16.3	7.12	9.27	31.67	**0.0002**
botttim	1,742	2,249	4.3	8.1	3.85	6.99	28.24	**0.0002**
asctim	1,853	2,373	13.2	21.3	8.00	11.11	39.50	**0.0002**
divetim	1,853	2,373	27.8	45.3	15.07	19.86	49.75	**0.0002**
descdist	1,853	2,373	6.9	11.0	6.02	7.32	15.14	**0.0016**
bottdist	1,742	2,249	0.7	1.6	1.08	1.91	28.24	**0.0002**
ascdist	1,853	2,373	6.8	10.8	5.97	7.21	15.39	**0.002**
bottdep.median	1,742	2,249	7.0	11.1	5.94	7.04	15.38	**0.0008**
maxdep	1,853	2,373	7.1	11.3	6.06	7.34	16.18	**0.0008**
postdive.dur	1,853	2,373	126.2	135.9	673.82	698.16	0.16	0.707
temp_sens_mean	1,853	2,373	4.9	2.5	1.13	1.05	53.11	**0.0002**
botfreq	1,742	2,249	0.2	0.2	0.14	0.15	2.46	0.129

## Discussion

Detailed knowledge of seabirds feeding ecology across the whole breeding season is essential to understand their time and energy allocation during breeding [86, 87]. To our knowledge foraging behaviour of little auks during the pre-laying period, has not been yet investigated, while it may be an important component of reproductive investments [19, 20, 87]. In this study combining GPS-TDR-tracking and remote sensing data we found sex differences in foraging habitat niches and some dive variables. We also found that little auks during the pre-laying period performed foraging trips with higher maximal range, utilized larger home ranges, and dived in colder water temperature compared to other phases of breeding.

### Foraging ecology during the pre-laying period

As we predicted, little auks during the pre-laying period performed foraging trips with a higher maximal range than later during the breeding period. This pattern is generally consistent with other seabirds, especially procellariforms [16, 25, 26]. However, direct comparison is not easy as, in contrast to the cited studies, our data were collected before the pre-laying exodus (Supplementary Materials Fig. S1) that occurs in little auks five days prior to egg-laying [51].

As we expected, distance from the colony to foraging locations and home range area were higher during the pre-laying period compared to the incubation period. Such a pattern has been also observed in another seabird, a procellariform, the thin-billed prion *Pachyptila belcheri* [28]. Closer distance to the colony and smaller home ranges during the incubation may be explained by necessity of regular returns to the colony to change incubating partner (in the little auk incubation bouts last ~ 12 h per day [88, 89]). In contrast to our expectations, however, distance from the colony to foraging locations and home range areas did not differ significantly between pre-laying and chick rearing periods. This discrepancy between expectations and obtained results could be explained by the fact that during the long foraging trips performed during the chick rearing period little auks may also reach distant foraging areas [42, 47, 70].

As we predicted, general foraging habitat preferences during the pre-laying period were similar to other phases of breeding. Studied birds mainly foraged in cold SST zone within the shallow sea depth shelf zone in eutrophic and enriched water regimes as in other phases of breeding [10, 41, 70]. Cold water masses in shallow areas are optimal conditions for cold-water copepod, *Calanus glacialis* CV stage [71, 75], the preferred by little auks [37, 90, 91]. The inter-phase consistency in core ranges is not surprising given the necessity of regular returns to the colony, even during the pre-laying period, and little auk foraging preferences.

Zooplankton biomass was the highest in Shelf-Eutrophic zone, the most frequently visited little auks foraging microhabitat. However, low values in many foraging locations in other zones with enhanced productivity, were not expected. It can be explained by the fact that the zooplankton model used predicts biomass of all zooplankton organisms without distinction into larger (preferred by little auks) and smaller prey items. Total zooplankton abundance in shelf zooplankton communities in the Hornsund area may be higher in Atlantic water masses compared to Arctic ones despite the fact that abundance of *Calanus glacialis* exhibits the opposite pattern with dominance in Arctic-origin water masses [72]. Thus, low zooplankton biomass does not always indicate low biomass of energy-rich Arctic copepods.

Some foraging locations were situated in Deep sea depth zone but regardless of sea depth zone little auks dived at similar depths. Mean maximal depths of dives recorded during the pre-laying period found in this study (9.4 m) were similar to these recorded during the chick-rearing period in Hornsund in 2018 [9.0 m (this study)] and in 2007 in Hornsund and two other colonies (one on Spitsbergen and one in Greenland; 9.9 m) [46]. Maximum diving depth recorded during the pre-laying period (34.2 m) was similar to the one recorded during the chick rearing period in Hornsund (40.7 (this study), 37.8 m [46]). All these findings suggest that 9–10 m is an optimal diving depth, perhaps related to the vertical copepods availability. Indeed, *Calanus glacialis* dominated over *Calanus finmarchicus* in -10 – 0 m depth layer in Hornsund area [92].

We found significantly lower bottom frequency during the pre-laying period reflecting more V-shaped dives (generally interpreted as ‘searching’ dives; e.g. [69]) than during the chick-rearing period. It suggests that birds during the chick rearing period were more concentrated on foraging in well known foraging areas. During the pre-laying period, not being obliged to return to the colony that often, little auks may explore various potential foraging grounds searching for prey. It cannot be excluded, however, that these differences reflects different food availability among the stages of the breeding season, as availability of various developmental stages of copepods varies temporarily and spatially. Monthly abundances of older developmental stages of cold-water copepod, *Calanus glacialis* (i.e. CV-VI stages) in waters in Svalbard coast have been characterized by a sharp and moderate peak in Atlantic waters in June, and a sharp peak with much higher abundances in July in the Arctic waters [93]. It could also explain a higher bottom frequency (i.e. indicating more U-shaped dives) during the peak of preferred prey abundance in July, i.e. during the chick-rearing period. Nevertheless abundance of *Calanus glacialis* (CV-VI stages) in Svalbard coastal waters in June (i.e., during little auks pre-laying period) in Arctic waters was relatively high, comparable to Atlantic waters being there the highest in this month [93].

### Sex differences

As expected, we found significant sex differences in several dive characteristics and foraging habitat niches. Dives of females were characterized by shorter duration of particular phases of dive, shorter distances covered during particular phases of diving, and shallower diving. Temperatures recorded by sensor during the bottom phase of diving were higher in females compared to males. Shorter distances and duration of dives may be interpreted in the context of energy expenditures of females. Recent study revealed that the cost of little auks diving is very high [34]. Production of the large egg (25–27% of dry body mass of female [48]) is expected to be costly for females. But little auk females can relatively easily acquire needed resources while foraging—considering foraging on energy rich Arctic copepods, and assuming 75% efficiency in the transformation of the consumed energy to the egg [48], females need a daily equivalent of two chick food portions to form the highly calorific yolk. Providing this level of energy resources does not seem to be a significant burden [48]. It cannot be excluded that sex differences in body mass and costs of egg production may be season-specific. In some years the costs of all parental activities may be comparable between the sexes, but in unfavourable foraging conditions, females may pay higher prices [48]. Thus, little auk females tracked in this study might not be able to allocate more energy to costly deeper diving. High initial reproductive investment is postulated as one of the possible reasons of brood desertion by female little auks [30].

Thus, wider foraging habitat niches and higher temperatures experienced by females during the bottom phase of diving may be interpreted in terms of energy constraint hypothesis, i.e. specific dietary requirements of females forming the egg. Some nutrients crucial to egg formation (as calcium and some fatty acids) may be difficult to obtain for little auk females regardless of the season, constituting a constraint in egg production [48, 94]. Acquisition of these nutrients may require foraging on specific prey, that inhabits specific microhabitats. A higher proportion of V-shaped dives compared to males may suggest foraging in different foraging habitat with specific prey. Indeed, foraging locations of one of the tracked females was situated in the deep water zone with higher water temperatures, which suggests foraging on some supplementary food associated with deep water zone, e.g. energy-rich *Calanus hyperboreus* preferring open sea deep water zone in West Spitsbergen area [71]. The older stages of this copepod (i.e. copepodite stages CV and CVI) were the most abundant in Atlantic waters close to Svalbard coast in June [93]. Nevertheless, the majority of foraging locations of both sexes were situated in cold water shelf zone representing optimal conditions for preferred little auks prey – *Calanus glacialis* [71, 72]. Given considerable inter-sex overlap in foraging ranges and niches, similar feeding trips duration and distances, the “intersexual competition” hypothesis suggesting one sex outcompeting another seems to be a less probable explanation of the observed differences in foraging ecology between males and females.

In contrast to our predictions, we did not find sex differences in foraging trip distances or duration. Also, home and core range areas did not differ significantly between the sexes. This lack of significant differences should be interpreted with caution as these results are based on low sample size (two females and four males).

Sex differences in foraging range and niches during the pre-laying period observed in this study have been also observed in some procellariforms. Usually males, often playing a greater role in nest site defence, forage more locally and return to the colony more often than females performing long distance trips (pre-laying exodus) to productive waters to gain resources for egg production (e.g. [16, 24, 28]). However, sometimes the opposite pattern has been found, e.g. in the Barau’s petrel *Pterodroma baraui,* with females foraging closer to the colony in less productive waters than males [13] or in the Chatham petrel *Pterodroma axillaris*, with a greater maximum range of males than females [95]. The studies of pre-laying activities in alcids are mainly focused on colony attendance [22, 23, 96–98], not on foraging trips, so it is not possible to compare them with our results. However, one may expect some inter-species differences driven by various factors like duration of the pre-laying exodus [23, 99] or the main site of copulation (in contrast to little auks in auklets and puffins copulation takes place at sea [100]; thus, for males of rhinoceros auklets *Cerorhinca monocerata*, staying together with mates at sea might be critical for successful copulation and mate-guarding [22]). Various patterns of sex differences in foraging ecology during the pre-laying period in particular species have been usually interpreted by specific parental roles in terms of energy constraint hypothesis, especially in the case of monomorphic species [13]. The inconsistency in the patterns of observed sex differences highlights the need to examine species carefully, as extrapolation of findings from one species to another may be inappropriate.

### Conservation implication

**Fig. 6:**
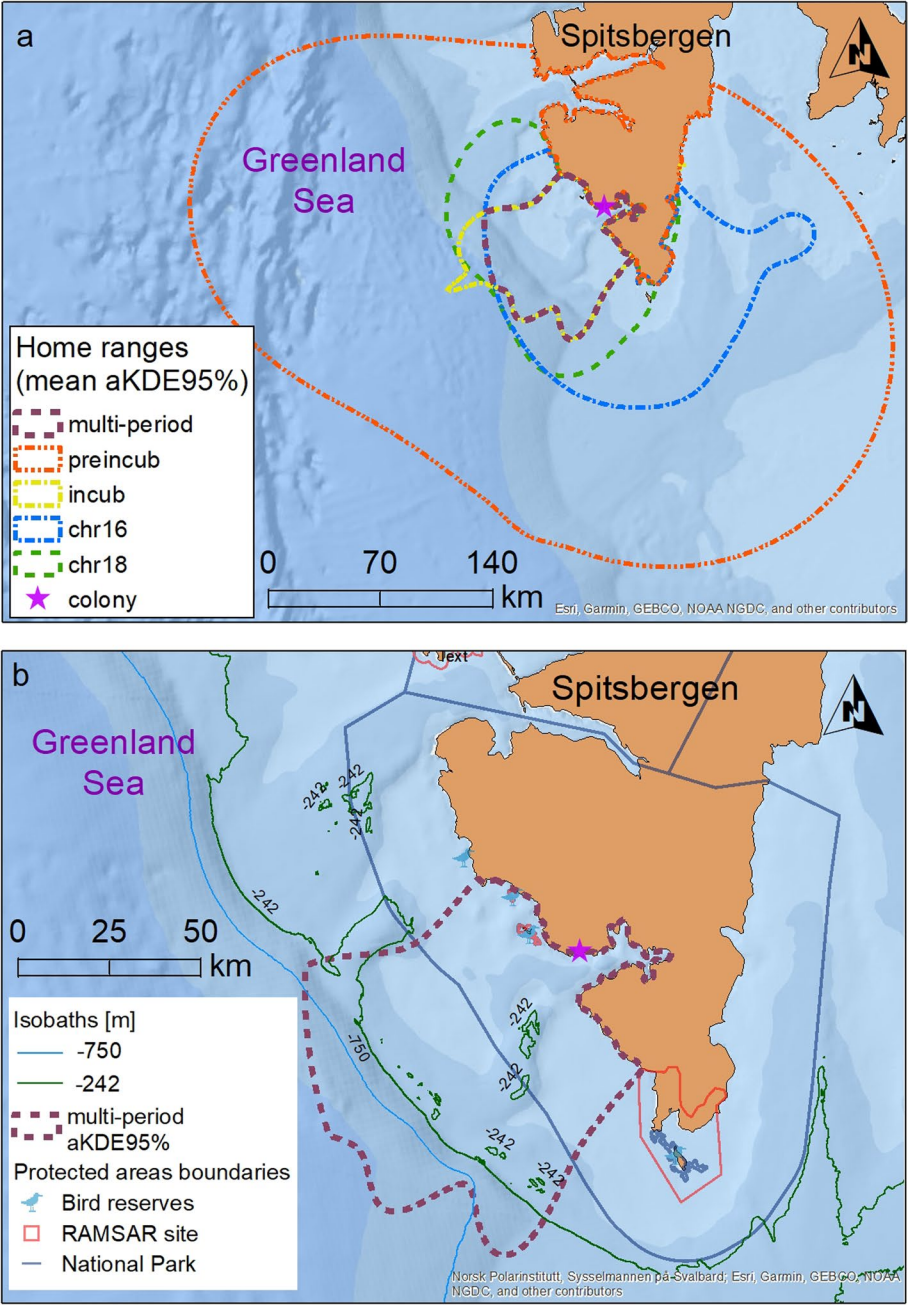
Area of home areas used by little auks from Hornsund colony (pink star). **a** Home areas utilized during the pre-laying (preincub, red), incubation (incub, yellow), and chick rearing period 2016 (chr16, blue) and 2018 (chr18, green) and multi-period overlap zone (dark brown). **b** Area of overlapping multi-period home areas (violet dashed line) and protected areas. Isobaths -242 m (green line) and -750 m (light blue line) divide the area into Shelf, Off-Shelf and Deep sea depth zones Background: The Arctic Ocean Base tile layer from Esri (https://services.arcgisonline.com/arcgis/rest/services/Polar/Arctic_Ocean_Base/MapServer); protected areas in b: Norwegian Polar Institute, Governor of Svalbard

Tracking data provide unparalleled information on the distribution of marine taxa which are important to effective conservation planning [101, 102]. The delineated area intersecting home ranges for pre-laying, incubation and chick-rearing periods, and thus constituting the area utilized during the whole breeding season, covers an area of 5,884 km^2^ and is located mainly in shallow sea depth shelf zone adjacent to the colony (Fig. 6). But it also includes Off-shelf and Deep sea depth zones. This area is partially located within boundaries of South Spitsbergen National Park and Marine Protected Area Svalbard West (covering marine part of South Spitsbergen National Park). Little auks multi-period home range includes also some other protected areas of high biodiversity level and conservation importance—some bird reserves and RAMSAR sites. It would be recommended to enlarge Marine Protected Area (MPA) Svalbard West at least to the limits of Shelf Zone, to cover the majority of the delineated area intensively utilized by little auks during the whole breeding season. The frontal zone over the shelf break with enhanced productivity and concentration of plankton is attractive foraging ground not only for little auks but also many other marine organisms (e.g. [103–105]). An effective protection of foraging areas as MPA may serve as an effective tool to conserve biodiversity and improve ecosystem functioning [106].

### Limitations of the study

Our study provides valuable findings on the foraging ecology of the little auk during the pre-laying period, a breeding phase so far neglected in the literature. Nevertheless, some limitations of our study should encourage further investigation.

First, our findings are based on a relatively small sample size despite considerable efforts we put in, and this is because of logistical constraints (to reach the study site early in the season and capture sufficient number of birds during the period when they are quite elusive is quite a challenge). Thus, some results should be interpreted with caution – e.g. lack of sex differences in core and home range area or distance to foraging grounds. While it is hard to overcome these constraints using the same field and methodological approach, future studies, even if again based on small sample size should aim to verify our findings on sex-differences in foraging habitat niches.

Second, our data did not cover the entire pre-laying period as we have no data from the last five days prior to egg laying (Supplementary Materials Fig. S1). In these few days before laying females presence in the colony is the lowest [51], meaning they spend this time outside of the colony. Thus, comparison with other studies, often conducted during the pre-laying exodus, should be treated with caution. However, it does not mean that the little auks females studied here did not gain resources for egg formation during the study period as yolk formation lasts 4–6 days [48], and further 2–4 days are needed to accumulate albumen and eggshell while the ovum is passing the oviduct [23, 99].

Third, our study is based on the behaviour of individuals with relatively heavy loggers. Although the weight of the device was acceptable given existing literature recommendations and we did not record atypical behaviour of tagged individuals, it cannot be excluded that time and/or energy budget of individuals could be altered to some extent. They could expend extra energy to forage having an extra burden and increased drag, and that could affect some aspects of flight and/or dive performance [107, 108]. However, as we compared the data from various stages of breeding from studies using the same type of loggers, one may expect the same potential bias in all years. Future studies should be performed based on more miniaturized devices, if available.

Finally, one needs to bear in mind that our comparison of the pre-laying period with other breeding phases was based on data collected in different years, with various environmental conditions. However, in the part of the Barents Sea, where we carried out the study, the water masses follow the bottom topography stabilizing the position of the Polar Front on the shelf break [58]. Thus, the general spatial pattern of colder and warmer water masses in the little auks foraging areas are relatively stable in time. Nevertheless, evaluation of the effect of environmental condition on the differences in little auk foraging ecology between the breeding phases clearly deserves further investigation.

## Conclusions

We demonstrated that little auks females during the pre-laying period explored wider foraging niches performing shorter and shallower dives compared to males. These differences may be attributed to sex-specific nutritional or/and energetical constraints at this stage of breeding (egg formation for females and nest site and paternity guarding for males). We also found that little auks dives in various sea depth zones were characterized by similar bottom depths and different temperatures. They explored generally the same areas and environmental conditions as during the subsequent phases of breeding. However, they had wider home ranges compared to the incubation period.

The results of this study emphasize the importance of shelf Arctic-type water masses as the foraging areas for little auks during successive stages of breeding. Knowledge about the foraging ecology of seabirds in the particular phases of breeding is crucial to understand carry-over effects between the stages of the reproduction period and to identify key foraging areas utilized by birds during the whole breeding season.

### Supplementary Information

**Supplementary Material 1. **

## Data Availability

Raw data are available from the corresponding author upon a reasonable request.
