# Supplementary material for: Sex differences in foraging ecology of a zooplanktivorous little auk Alle alle during the pre-laying period: insights from remote sensing and animal-tracking
Source: Front Zool. 2024 Apr 17;21:12. doi: 10.1186/s12983-024-00534-2 (PMC11022494; doi:10.1186/s12983-024-00534-2)
Supplement: Supplementary file 1 — Supplementary Material 1. [file 12983_2024_534_MOESM1_ESM.docx]

Supplementary materials

**Sex differences in foraging ecology of a zooplanktivorous little auk *Alle alle* during the pre-laying period: Insights from remote sensing and animal-tracking**

Dariusz Jakubas*, Katarzyna Wojczulanis-Jakubas, Lech Marek Iliszko

Department of Vertebrate Ecology and Zoology, Faculty of Biology, University of Gdańsk, 80-308 Gdańsk, Poland

**Table S1.** The number of analysed trips of GPS-tracked individuals with their basic statistics.

Grey fields – females, other fields – males, n = number of GPS fixes


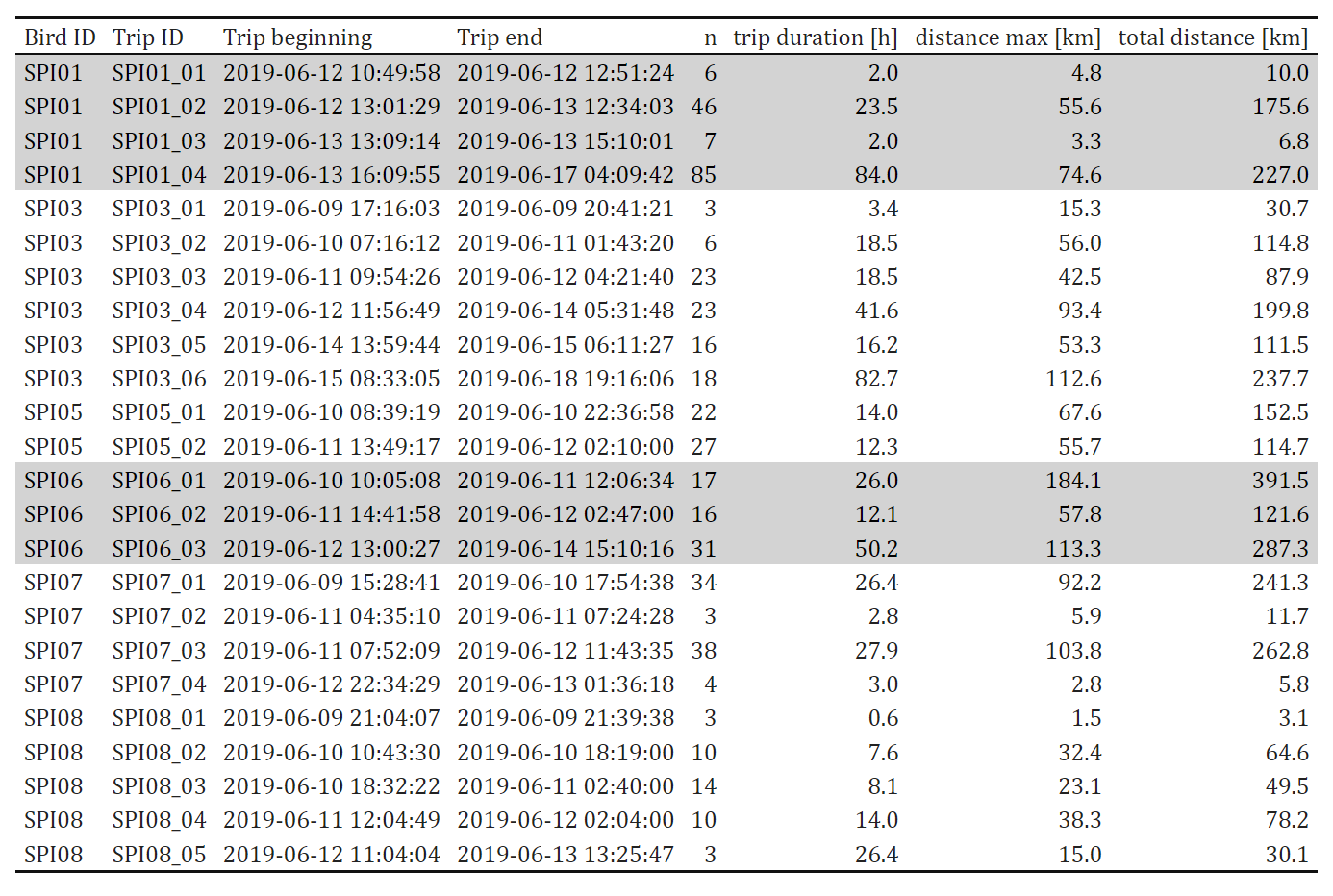


**
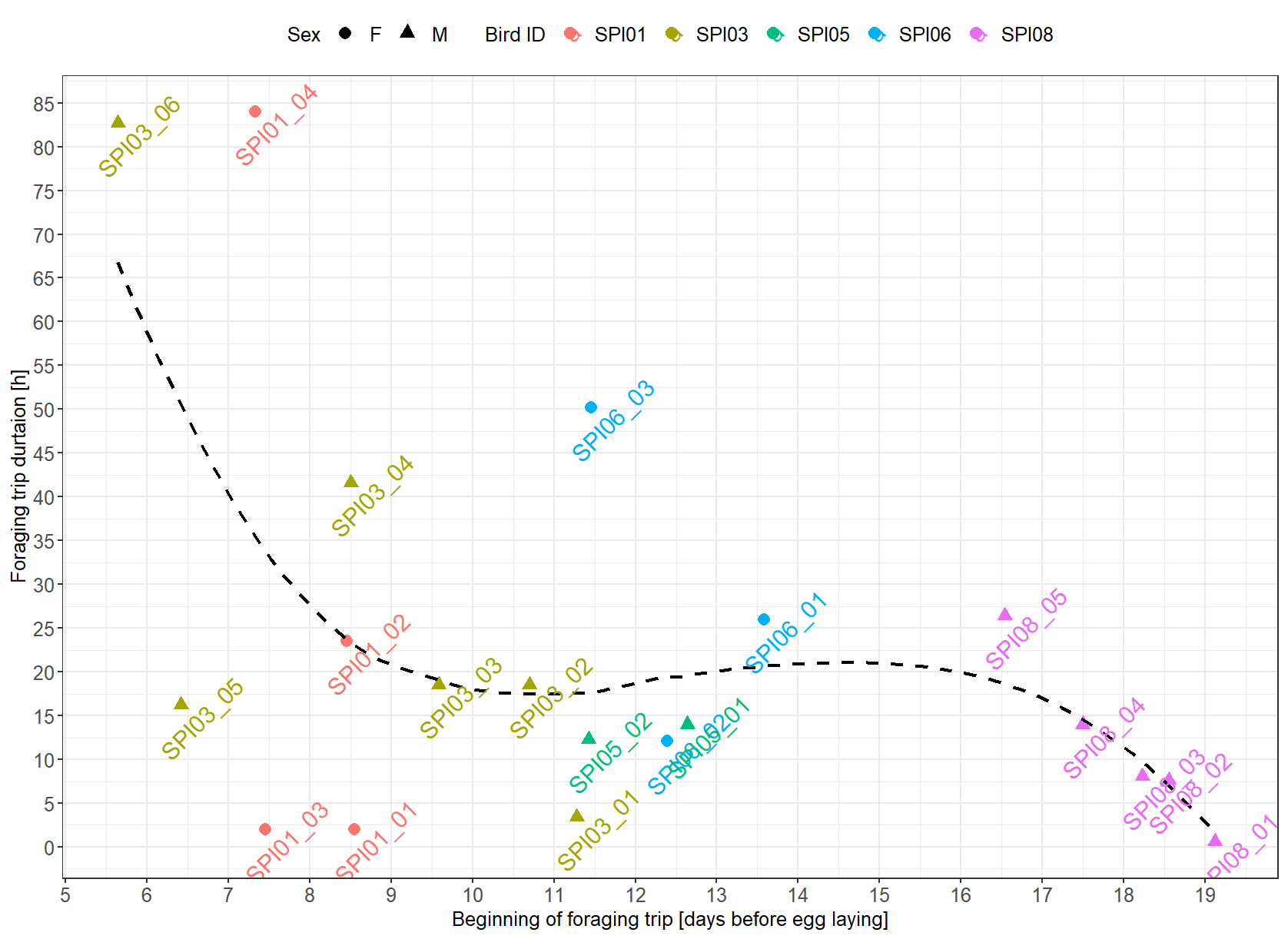
**

**Fig. S1.** Timing of foraging trips performed by GPS-tracked little auks breeding in Hornsund (SW Spitsbergen) during the pre-laying period. Smooth curve (black dashed line) fitted by local polynomial regression (loess). Point labels – foraging trip identity.


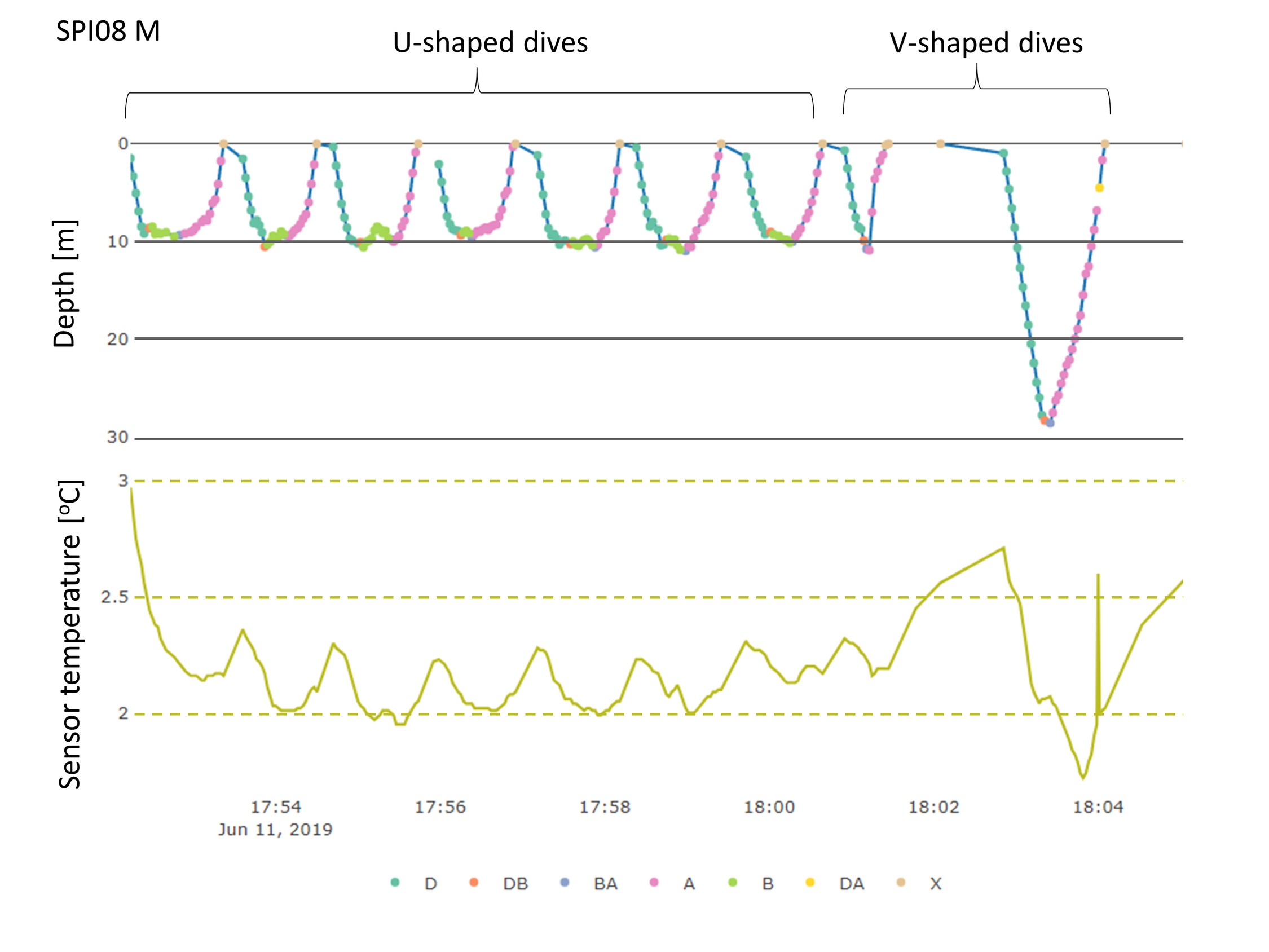


**Fig. S2.** An example of depth and temperature sensor records of individual SPI08, with two types of dives (U- and V-shaped; see description in the main text). Phases of dive: descent=D, descent/bottom=DB, bottom/ascent=BA, ascent=A, X=surface.


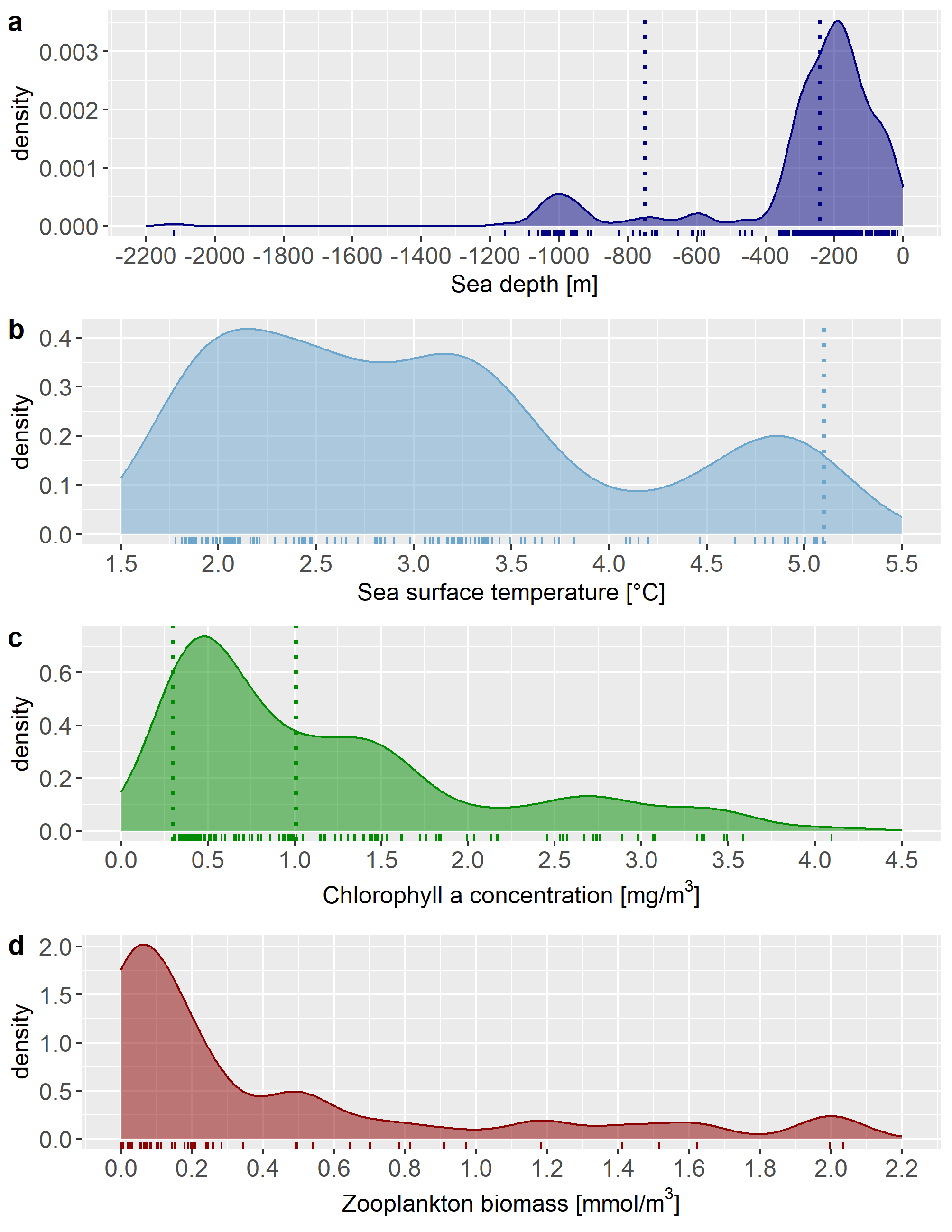


**Fig. S3.** Density plots of environmental conditions (A. Sea depth; B. Sea surface temperature; C. Chlorophyll a a concentration; D. Zooplankton biomass) in foraging locations of GPS-tracked little auks breeding in Hornsund (SW Spitsbergen) during the pre-laying period. Rug plots show point distribution. Vertical isolines indicate thresholds distinguishing various zones: A. Sea depth zones: Deep sea zone < -750 m, Off-shelf zone: -750 - -242 m, Shelf zone: > -242 m , B. Sea surface temperture zones: Cold: 0-5.1 ^o^C, Transient > 5.1 ^o^C, C. Productivity regimes: Eutrophic: 0.3-1 mg/m^3^, Enriched waters: > 1 mg/m^3^.


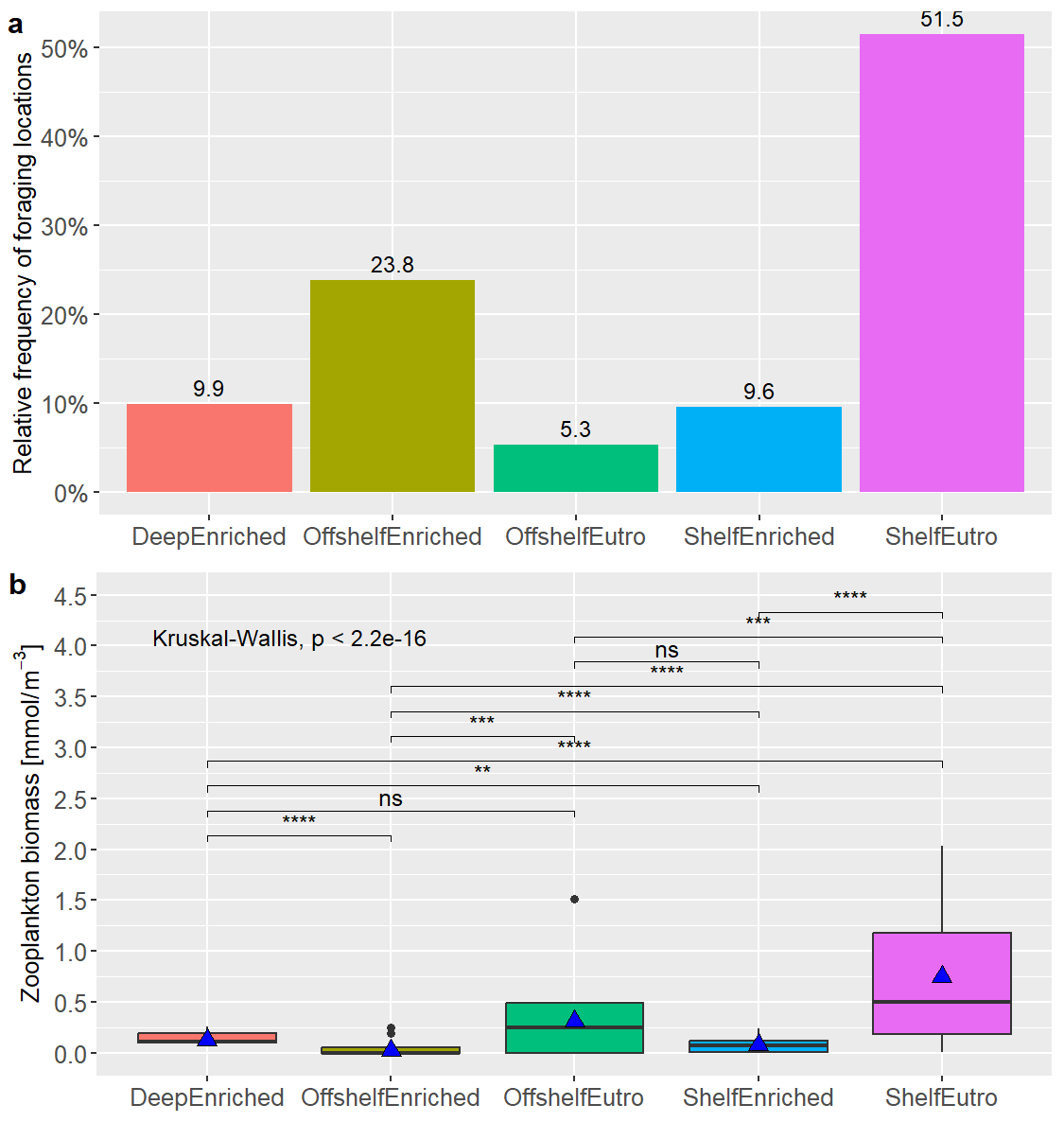


**Fig. S4.** Relative frequency of foraging locations of little auks breeding in Hornsund (SW Spitsbergen) during the pre-laying period in particular Depth-Productivity zones (A) and zooplankton biomass in these zones (B). Boxplots show the median (band inside the box), the first (25 %) and third (75 %) quartile (box), the lowest and the highest values within 1.5 interquartile range (whiskers), and outliers (dots). Blue triangles indicate mean values. Horizontal lines above boxplots in Panel B show results of Wilcoxon test (p values: ns: p > 0.05, *: p <= 0.05, **: p <= 0.01, ***: p <= 0.001, ****: p <= 0.0001).


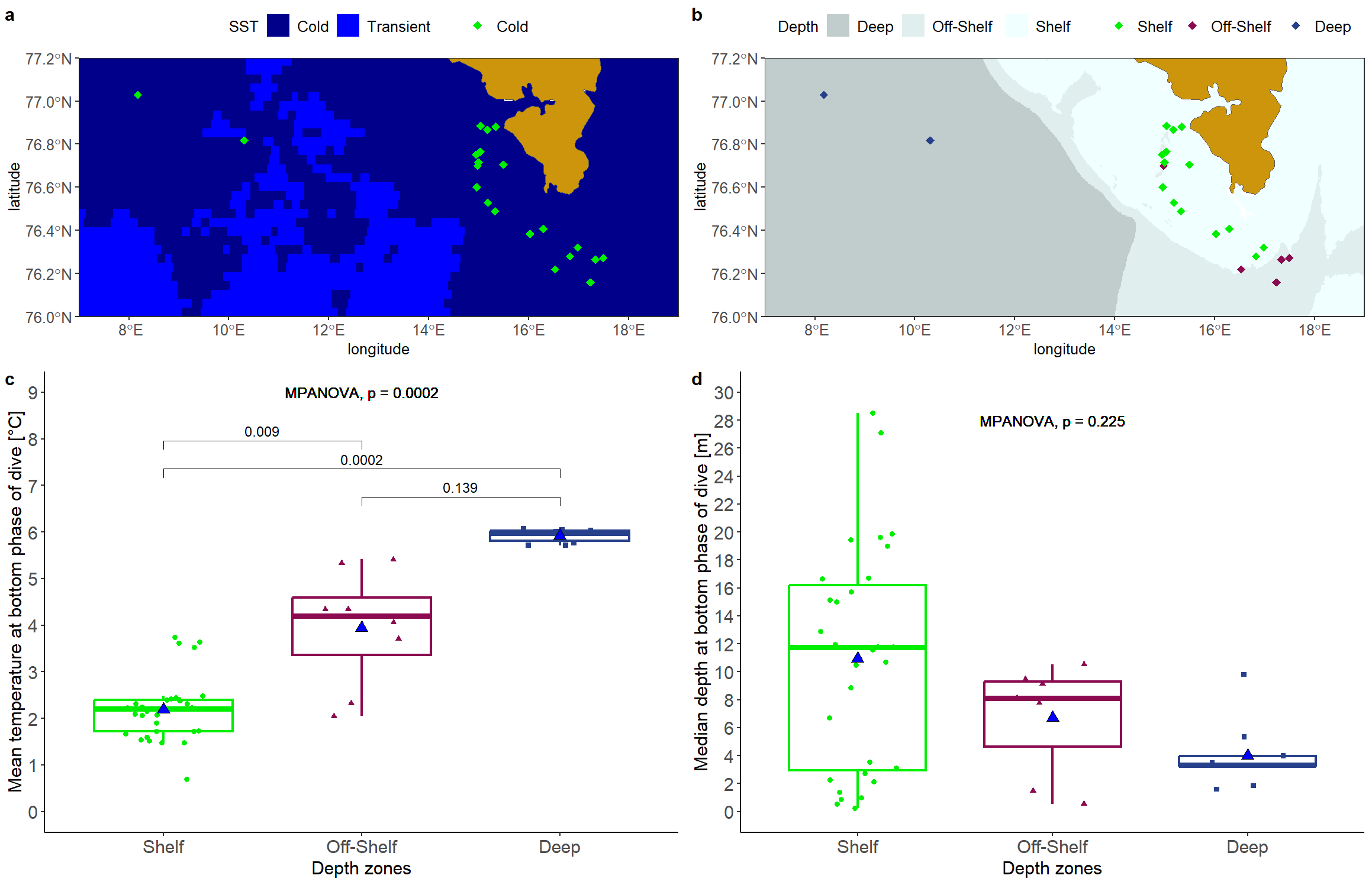


**Fig. S5.** Characteristics of dives performed by little auks in sea surface and sea depth zones. A. map of sea surface temperature zones and locations of little auks dives in particular zones (points). B. map of sea depth zones and locations of little auks dives in particular zones (points) C. boxplots of mean sensor temperature during the bottom phase of dive in particular sea depth zones. D. boxplots of median diving depth during the bottom phase. Boxplots show the median (band inside the box), the first (25%) and third (75%) quartile (box), the lowest and the highest values within 1.5 interquartile range (whiskers) and outliers (circles). Blue triangles indicate mean values.

**Table S2** Niche size for particular individuals and probabilities of individual A to be found within the foraging habitat niche of individual B (alpha = 95%). ^f^ – females, ^m^ -males. * - pair members from the same nest.

|  | SPI01^f^* | SPI03^m^* | SPI05^m^ | SPI06^f^ | SPI07^m^ | SPI08^m^ |
| --- | --- | --- | --- | --- | --- | --- |
| Niche size | 12,023.95 | 1,222.04 | 68.35 | 7,630.44 | 584.79 | 35.97 |
| Probability | Ind B |  |  |  |  |  |
| IndA | SPI01 ^m^* | SPI03 ^m^* | SPI05^m^ | SPI06^f^ | SPI07^m^ | SPI08^m^ |
| SPI01^f^* | - | 21.50 | 1.46 | 36.27 | 4.96 | 0.44 |
| SPI03^m^* | 96.78 | - | 12.96 | 56.17 | 17.24 | 3.87 |
| SPI05^m^ | 99. 76 | 99.02 | - | 67.16 | 47.54 | 0.63 |
| SPI06^f^ | 52.01 | 11.36 | 0.77 | - | 12.93 | 0.12 |
| SPI07^m^ | 61.67 | 22.36 | 4.85 | 91.04 | - | 0.78 |
| SPI08^m^ | 97.89 | 93.74 | 5.10 | 31.31 | 23.09 | - |


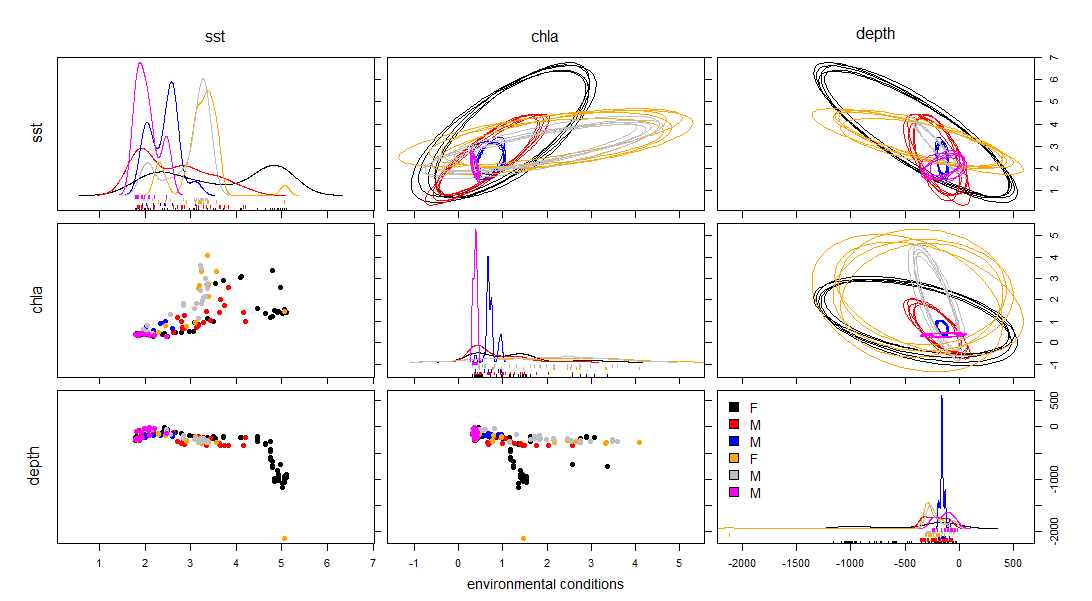


**Fig. S6.** Foraging habitat niches [expressed by sea surface temperature (sst), chlorophyll a concentration (chla) and sea depth [m] in foraging locations of GPS-tracked individuals) of particular individuals (marked with different colours; M – males, F - females) of little auks breeding in Hornsund (SW Spitsbergen) during the pre-laying period. Distribution of particular habitat variables are shown in one-dimensional density plots and two-dimensional scatterplots. Ellipses represent ten random projections of the foraging niches in two-dimensional perspectives of two variables.
